# Lactic acid drives NLRP3 inflammasome activation and caspase-1–like cytokine cleavage via intracellular acidification

**DOI:** 10.1038/s41419-026-08708-y

**Published:** 2026-04-03

**Authors:** Hsin-An Lin, Hsin-Chung Lin, Ming-Hang Tsai, Yu-Jen Chen, Bo-Ying Bao, Kuen-Jou Tsai, Chieh-Tien Shih, Jau-Song Yu, Kun-Yi Chien, Kuo-Yang Huang, David M. Ojcius, Lih-Chyang Chen

**Affiliations:** 1 Department of Internal Medicine, School of Medicine, College of Medicine, National Defense Medical University, Taipei, Taiwan; 2 Division of Infectious Diseases and Tropical Medicine, Department of Internal Medicine, Tri-Service General Hospital, National Defense Medical University, Taipei, Taiwan; 3 Department of Internal Medicine, Songshan Branch of Tri-Service General Hospital, National Defense Medical University, Taipei, Taiwan; 4 Department of Pathology, School of Medicine, College of Medicine, National Defense Medical University, Taipei, Taiwan; 5 Division of Clinical Pathology, Department of Pathology, Tri-Service General Hospital, Taipei, Taiwan; 6 Department of Medical Research, MacKay Memorial Hospital, New Taipei City, Taiwan; 7 Department of Radiation Oncology, MacKay Memorial Hospital, Taipei, Taiwan; 8 School of Medicine, College of Medicine, MacKay Medical University, New Taipei City, Taiwan; 9 Department of Artificial Intelligence and Medical Application, MacKay Junior College of Medicine, Nursing and Management, Taipei, Taiwan; 10 Department of Medical Research, China Medical University Hospital, Taichung, Taiwan; 11 Department of Pharmacy, China Medical University, Taichung, Taiwan; 12 Department of Laboratory Medicine, MacKay Memorial Hospital, Taipei, Taiwan; 13 Department of Nursing, College of Nursing, MacKay Medical University, New Taipei City, Taiwan; 14 Molecular Medicine Research Center, Chang Gung University, Taoyuan, Taiwan; 15 Graduate Institute of Biomedical Sciences, College of Medicine, Chang Gung University, Taoyuan, Taiwan; 16 Department of Otolaryngology-Head and Neck Surgery, Chang Gung Memorial Hospital at Linkou, Taoyuan, Taiwan; 17 Research Center for Food and Cosmetic Safety, College of Human Ecology, Chang Gung University of Science and Technology, Taoyuan, Taiwan; 18 Department of Biochemistry and Molecular Biology, Chang Gung University, Taoyuan, Taiwan; 19 Clinical Proteomics Core Laboratory, LinKou Chang Gung Memorial Hospital, Taoyuan, Taiwan; 20 Graduate Institute of Pathology and Parasitology, College of Medicine, National Defense Medical University, Taipei, Taiwan; 21 Department of Biomedical Sciences, University of the Pacific, Arthur Dugoni School of Dentistry, San Francisco, CA USA; 22 Institute of Biomedical Science, College of Medicine, MacKay Medical University, New Taipei City, Taiwan; 23 Department of Optometry, College of Medicine, MacKay Medical University, New Taipei City, Taiwan

**Keywords:** Inflammasome, Interleukins

## Abstract

Glycolysis is critical for NLRP3 inflammasome activation, yet the link between lactic acid metabolism and inflammasome signaling remains unclear. Here, we show that stimulation of macrophages with the NLRP3 activators nigericin or ATP induces lactic acid production and efflux via a lactate dehydrogenase–dependent pathway. Accumulation of intracellular lactic acid leads to cytoplasmic acidification, which promotes NLRP3 inflammasome activation. Concurrently, elevated extracellular lactic acid impairs lactate efflux, exacerbating intracellular acidification and amplifying ASC speck formation, caspase-1 activation, and IL-1β secretion. Alkalinization of the extracellular milieu prevents intracellular acidification and abolishes inflammasome activation. Mechanistically, intracellular lactic acidification promoted mitochondrial dysfunction and reactive oxygen species production, and concurrently induced phosphorylation of the stress kinase PKR, which facilitated PKR–NLRP3 interaction and inflammasome assembly through parallel pathways. Independently of inflammasome signaling, lactic acid also directly cleaves pro-IL-1β and pro-IL-18 into mature forms through a mechanism requiring its carboxyl group and mimicking caspase-1 substrate specificity. Mass spectrometry analysis revealed lactic acid–mediated cleavage of pro-IL-1β at Asp116, the canonical caspase-1 site. In a murine model of polymicrobial sepsis induced by cecal ligation and puncture, systemic lactate administration exacerbated inflammation, increased IL-1β levels and neutrophil infiltration, induced hypothermia, and worsened survival. Together, these findings identify intracellular lactic acidification as a metabolic signal that promotes inflammation predominantly through NLRP3 inflammasome activation, while also revealing a potential inflammasome-independent cytokine processing mechanism under conditions of severe metabolic stress.

## Introduction

The NLRP3 inflammasome is a key innate immune sensor activated by pathogen- and damage-associated molecular patterns (PAMPs and DAMPs), such as nigericin and ATP [1]. Upon activation, NLRP3 assembles with the adaptor ASC and pro–caspase-1 to form a cytosolic complex that activates caspase-1. Activated caspase-1 cleaves pro–IL-1β and pro–IL-18 into their mature, secretable forms. Notably, cleavage of pro–IL-1β not only permits its release but also markedly increases its binding affinity to the IL-1 receptor, which is essential for downstream signaling [2]. In contrast, uncleaved pro–IL-1β is biologically inactive and unable to engage the receptor [2]. While NLRP3 inflammasome activation is vital for host defense, its dysregulation contributes to diverse inflammatory diseases, including sepsis [3]. In murine models, genetic ablation of inflammasome components confers protection against septic shock [4–8]. NLRP3 activation occurs rapidly—within minutes of stimulation [9]—and is triggered by intracellular perturbations in ion flux, mitochondrial integrity, and lysosomal function [1]. However, the molecular mechanisms linking these signals to NLRP3 activation remain incompletely understood.

Recent studies have identified glycolysis as a key metabolic driver of NLRP3 inflammasome activation. Upon NLRP3 stimulation, macrophages rapidly enhance glycolytic flux to meet the increased metabolic demands associated with inflammasome assembly and cytokine secretion [10]. Inhibition of glycolysis—through glucose deprivation or pharmacological blockade of key enzymes such as hexokinase, pyruvate kinase M2 (PKM2), or lactate dehydrogenase (LDH)—markedly suppresses inflammasome activation [4, 10, 11]. Conversely, enhancing glycolytic flux by blocking mitochondrial pyruvate uptake—via genetic deletion of mitochondrial pyruvate carrier 2 (MPC2)—further potentiates NLRP3 activation [10]. These observations suggest that glycolysis-derived metabolites, particularly lactic acid—the terminal product of aerobic glycolysis—may directly modulate inflammasome activity. Protein kinase R (PKR), a serine/threonine kinase activated by viral infection and metabolic stress [12, 13], has been shown to be essential for NLRP3 inflammasome activation [14]. Our previous work demonstrated that LDH inhibition reduces PKR phosphorylation and that pharmacological inhibition of PKR impairs NLRP3 inflammasome assembly and IL-1β secretion in macrophages [10]. However, the molecular mechanisms that connect glycolytic activity, lactic acid metabolism, and PKR-dependent inflammasome activation remain poorly understood.

Lactic acid, historically regarded as a metabolic byproduct of anaerobic glycolysis, accumulates not only during strenuous exercise [15] but also in various pathological contexts, including sepsis [16, 17], rheumatoid arthritis [18, 19], and cancer [20, 21]. Lactic acidosis represents one of the most common causes of acute metabolic acidosis in clinical settings [22]. Beyond its role in metabolism, lactic acid has emerged as a modulator of immune responses, exerting both pro- and anti-inflammatory effects depending on context, and thereby influencing disease progression [23, 24]. Notably, prolonged exposure to lactic acid under acidic conditions induces cleavage of pro–IL-1β. However, the resulting cleaved product migrates at ~20 kDa—distinct from the canonical 17 kDa mature form [4]—suggesting the involvement of a noncanonical, possibly chemical, cleavage mechanism.

In this study, we identify lactic acid as a metabolic driver of inflammation via both NLRP3 inflammasome–dependent and –independent pathways. Intracellular lactic acid promotes cytoplasmic acidification and NLRP3 activation, while extracellular lactic acid enhances this effect by blocking lactate efflux. Acidification facilitates PKR–NLRP3 interaction, and lactic acid also directly cleaves pro–IL-1β and pro–IL-18 in a caspase-1–like manner. In a murine sepsis model, lactate administration exacerbates inflammation and mortality. These findings highlight lactic acid as a dual-acting pro-inflammatory metabolite and potential therapeutic target.

## Materials and methods

### Reagents

Phorbol myristate acetate (PMA), nigericin, ATP, lactic acid (LA), sodium lactate (NaL), sodium chloride (NaCl), 3-hydroxybutyric acid (3-OBA), poly(dA:dT), and acetic acid (AcOH) were acquired from Sigma-Aldrich. Hydrochloric acid was obtained from Merck, and 3-chloro-5-hydroxybenzoic acid (3Cl-5OH-BA) and MCC950 were purchased from MedChemExpress.

### Cell culture

Mouse bone marrow-derived macrophages (BMDMs) were generated from bone marrow cells, which were collected from the tibias and femurs of C57BL/6 J mice by flushing with cold PBS using a 25-G needle. The cells were cultured in DMEM medium supplemented with 10% FCS and 10 ng/ml M-CSF (PeproTech) for 8 days. The THP-1 (human leukemia monocytic) cell line was purchased from the Biosource Collection and Research Center, Food Industry Research and Development Institute (Hsinchu, Taiwan). ASC-mCherry-expressing THP-1 cells and ASC-shRNA-expressing THP-1 cells were described previously [25, 26]. All THP-1 cells were maintained in RPMI as described previously [27]. For macrophage differentiation, THP-1 cells were stimulated with 200 nM PMA for 20 h [27]. For NLRP3 inflammasome activation, the cells were treated with 10 μM nigericin for 45 min and 5 mM ATP for 1 h. For AIM2 inflammasome activation, cells were transfected with poly(dA:dT) for 4 h using Lipofectamine 2000 (Invitrogen) as described previously [28]. To examine the effect of external lactic acid, the cells were treated with LA and NaL at indicated concentrations and immediately followed inflammasome stimulation. To examine the effect of external pH, the cells were treated with HCl and NaOH at indicated pH and immediately followed inflammasome stimulation. For lactic acid fermentation inhibition, GSK2837808A (MedChemExpress, 10 μM) was applied for 1 h prior to inflammasome stimulation. For pan-caspase inhibition and caspase-1-specific inhibition, THP-1-derived macrophages were treated with Z-VAD-FMK (MedChemExpress, 20 μM), or Y-VAD-FMK (MedChemExpress, 20 μM) for 1 h. For GPR81 activation or inhibition, cells were treated with 3Cl-5OH-BA (1 mM) or 3-OBA (15 mM) for 1 h. For knockdown of NLRP3 and caspase-1, THP-1-derived macrophages were transfected with siRNA specifically against NLRP3 and caspase-1 using Lipofectamine 2000 (Invitrogen) as described previously [28]. For overexpression of pro-IL-1β, HEK293T cells were maintained with DMEM and transfected with pLAS2w.Pneo-pro-IL-1β-FLAG and pLAS2w.Pneo empty plasmid using Lipofectamine 2000 as described previously [29].

### Mouse cecal ligation and puncture

All animal procedures were approved by the Institutional Animal Care and Use Committee of MacKay Medical University (A1090020(m)-6) and performed in accordance with ARRIVE 2.0 guidelines. C57BL/6 mice (National Laboratory Animal Center, Taiwan) were anesthetized with 2% isoflurane and subjected to cecal ligation and puncture (CLP) to induce polymicrobial sepsis. The cecum was exteriorized via a left abdominal incision, ligated 1 cm from the tip using 4-0 silk suture, and punctured once with a 28-gauge needle. A small amount of fecal material was extruded to ensure patency, and the cecum was returned to the peritoneal cavity. The abdominal wall was closed with surgical clips. At 30 min post-CLP, mice were randomly assigned to receive a subcutaneous injection of 600 µl of either 154 mM sodium lactate or 154 mM sodium chloride, and/or an intraperitoneal injection of PBS or MCC950 (50 mg/kg). Blood was collected from the orbital sinus 6 h after CLP for measurement of plasma lactate and IL-1β. In parallel, whole blood was collected by retro-orbital bleeding into heparinized capillary tubes and immediately analyzed for blood pH and sodium concentration using a GEM Premier 4000 blood gas analyzer (Werfen). Additional blood samples were obtained by cardiac puncture, allowed to clot at room temperature, and centrifuged to isolate serum. Serum osmolarity was measured using a freezing-point depression osmometer (OSMO STATION OM-6060, ARKRAY). Peritoneal lavage fluid was collected for IL-1β, and peritoneal exudate cells were stained for flow cytometry. Neutrophils were identified as CD45⁺CD11b⁺Ly6G⁺ using a CytoFLEX S flow cytometer (Beckman Coulter). Antibodies used included CD45-APC (BioLegend, #103112), CD11b-BV605 (BioLegend, #101257), and Ly6G-PE (BD Biosciences, #551461). Gating strategy followed a previously described protocol with minor modifications [30]. For long-term studies, mice received a second dose of the assigned treatment 6 hours post-CLP, followed by twice-daily dosing for 5 consecutive days. Rectal temperatures were measured using digital rectal thermometers (CENTER TECHNOLOGY CORP., #CENTER301), and survival was monitored twice daily for the duration of the study.

### Immunoblot analysis

Experiments were performed according to our previously described protocol with some modifications [11]. Cells were lysed using RIPA buffer containing 50 mM Tris-HCl (pH 7.5), 150 mM NaCl, 10 mM MgCl_2_, 1 mM EDTA, and 1% Igepal CA-630, along with a protease inhibitor cocktail comprising 4.76 μg/mL leupeptin, 3.25 μg/mL aprotinin, 0.69 μg/mL pepstatin, and 1 mM phenylmethylsulfonyl fluoride, on ice for 30 minutes. To evaluate the maturation of IL-1β and the activation of caspase-1, culture supernatants were collected and mixed with one-tenth volume of 100% (w/v) trichloroacetic acid, followed by incubation at 4°C for 10 minutes. The resulting protein samples were separated through sodium dodecyl sulfate-polyacrylamide gel electrophoresis and transferred to polyvinylidene difluoride membranes (Millipore). The membranes were then incubated with specific primary antibodies, and subsequently with HRP-conjugated secondary antibodies. The primary antibodies used were directed against NLRP3 (Adipogen, #AG-20B-0014-C100, 1:1000), ASC (Santa Cruz, #sc-22514, 1:1500), IL-1β (Santa Cruz, #sc-32294, 1:1500), pro-caspase-1 (Cell Signaling, #2225, 1:1000), cleaved caspase-1 (Cell Signaling, #3866, 1:1000), PKR (Abcam, #ab226819, 1:1000), IL-18 (MBL, #D043-3, 1:1000), anti-FLAG (Merck, #SI-F1804, 1:3500), and GAPDH (Santa Cruz, #sc-32233, 1:10000). Immunoreactive bands were visualized using either a regular enhanced chemiluminescence (ECL) substrate (Clarity™ Western ECL Substrate, Bio-Rad) or a sensitive ECL substrate (Immobilon ECL Ultra Western HRP Substrate, Millipore). Immunoblot images were quantified using ImageJ software.

### Immunoprecipitation assay

For immunoprecipitation of PKR complex, THP-1-derived macrophages were lysed using RIPA buffer containing protease inhibitors as described above and immunoprecipitated with anti-PKR antibodies (Abcam, #ab226819) for overnight at 4 °C. The samples were then precipitated with PureProteome™ Protein G Magnetic Bead (Merck, #LSKMAGG10) for 1 h at 4 °C. For purification of pro-IL-1β-FLAG protein, cell extracts from HEK293T cells transfected with pLAS2w.Pneo-pro-IL-1β-FLAG plasmids expressing either wild-type (WT) or Asp116 mutants (D116A, D116N) of pro-IL-1β—obtained from the National RNAi Core Facility, Academia Sinica (Taipei, Taiwan)—were subjected to immunoprecipitation using anti-FLAG® M2 Magnetic Beads (Merck, #M8823) overnight at 4 °C. The immunoprecipitated proteins were then collected and analyzed by immunoblotting as described above.

### Cell-free inflammasome activation system

To measure inflammasome activation in cell-free condition, the levels of active caspase-1 and mature IL-1β in cell extracts, mature IL-1β in purified pro-IL-1β protein, and mature IL-18 in purified pro-IL-18 protein were detected by immunoblot analysis after indicated in vitro treatment. The cell extracts were prepared from THP-1-derived macrophages and HEK293T cells with or without siRNA knockdown or pro-IL-1β overexpression by RIPA lysis buffer as described above. The pro-IL-1β-FLAG protein was purified from cell extracts of pro-IL-1β-FLAG-overexpressing HEK293T cells by immunoprecipitation as described above. Recombinant pro-IL-1β and recombinant pro-IL-18 proteins were purchased from PeproTech. The in vitro treatment was conducted as follows: the cell extracts and purified pro-IL-1β and pro-IL-18 proteins were treated with LA (30 mM or as indicated), NaL (30 mM), HCl (pH 3.4), AcOH (30 mM), or recombinant active caspase-1 protein (Abcam, #ab39901, 0.02 unit/μl) in RIPA buffer for 30 min at room temperature. After in vitro treatment, the products were collected for immunoblot analysis as described above.

### IL-1β enzyme-linked immunosorbent assay

Cell culture supernatants or mouse peritoneal lavage fluids were assayed for human IL-1β and mouse IL-1β (eBioscience), respectively, as described previously [10].

### ASC speck-forming assay

The ASC-mCherry speck-forming cells were distinguished by flow cytometry as described by Sester et al. [31]. Briefly, nigericin-treated THP-1-ASC-mCherry cells were gated, analyzed for their inflammasome activation state by the analysis of pulse width to pulse area profile (W:A), and sorted for the presence/absence of ASC-mCherry specks.

### pHrodo assay

To analyze cytosolic pH, THP-1-derived macrophages were pretreated with Z-VAD-FMK for 1 h as described above and labeled with pHrodo Green (Thermo, #P35373) for 30 min in phenol red-free RPMI according to the manufacturer’s instructions.

According to experimental requirements, THP-1-derived macrophages were treated with GSK2837808A, LA, NaL, HCl, or NaOH as described above, followed by nigericin stimulation for 8 minutes. The cells were analyzed by flow cytometry (excitation/emission: 509/533 nm) using a CytoFLEX S flow cytometer (Beckman Coulter). Mean fluorescence intensity (MFI) was calculated using CytExpert software, version 2.6.

### Sample preparation and in-gel collection

Recombinant pro-IL-1β protein (Sino Biological, #10139-H07E) was incubated with either 30 mM lactic acid (Sigma, #L6661-100ML) or 0.02 U/μL of active recombinant human caspase-1 (Abcam, #ab39901) in RIPA buffer for 15 minutes at room temperature. Following the reaction, samples were separated by 12% SDS-PAGE and stained with 0.5% Coomassie Brilliant Blue G-250 (AppliChem GmbH, Darmstadt, Germany). The corresponding gel lanes were excised and subjected to in-gel chymotryptic digestion.

### N-terminal dimethylation labeling and in-gel protein digestion

Gel pieces were destained with Buffer A (30 mM ammonium bicarbonate [NH₄HCO₃], 40% acetonitrile [ACN]) for 10 minutes at room temperature and washed with Buffer B (30 mM triethylammonium bicarbonate, 40% ACN). Samples were then dehydrated in ACN (Mallinckrodt Baker) and dried using a SpeedVac. Proteins were reduced in 25 mM NH₄HCO₃ containing 10 mM dithiothreitol (Biosynth AG) at 56 °C for 60 minutes, followed by alkylation in 55 mM iodoacetamide (Amersham Biosciences) at room temperature for 45 minutes in the dark. After alkylation, gel pieces were washed with Buffer B, dehydrated in ACN, and dried again using a SpeedVac. For N-terminal dimethylation labeling, samples were incubated at room temperature for 20 minutes in a labeling buffer containing 120 mM sodium cyanoborohydride, 0.8% formaldehyde, and 168 mM sodium acetate (pH 5.2) [32]. After labeling, samples were washed with Buffer A, dehydrated in ACN, and dried using a SpeedVac. Proteins were digested overnight at 37 °C with chymotrypsin (Thermo Fisher, #90056). Peptides were extracted with 1% trifluoroacetic acid (TFA) in 40% ACN and subsequently dried in a SpeedVac.

### LC-MS/MS analysis

Peptides were reconstituted in 0.1% formic acid and analyzed using a nano-LC system coupled to an LTQ-Orbitrap hybrid mass spectrometer (Thermo Fisher Scientific, CA, USA), as previously described [33]. Samples were first loaded onto a trap column (Zorbax 300SB-C18, 0.3 × 5 mm; Agilent Technologies) at a flow rate of 20 μL/min in 0.1% formic acid and then separated at a flow rate of 0.2 μL/min on a 10 cm analytical BEH C18 column (Waters, 130 Å pore size, 1.7 μm, 100 μm × 100 μm) with a 20 μm emitter tip (New Objective, Woburn, MA, USA). Peptides were eluted with a linear gradient as follows: 0–10% buffer B (99.9% ACN with 0.1% formic acid) for 3 min, 10–30% buffer B for 35 min, 30–35% for 4 min, 35–50% for 1 min, 50–95% for 1 min, and 95% buffer B for 8 min, at a flow rate of 0.25 μL/min. Orbitrap resolution was set to 30,000, and the ion signal of (Si(CH₃)₂O)₆H⁺ at m/z 445.120025 was used as the lock mass for internal calibration. Data-dependent acquisition was used, consisting of one full MS scan followed by six MS/MS scans of the top 6 most intense precursor ions, with dynamic exclusion for 180 seconds. The MS scan range was m/z 400–2000. For MS/MS scans, spectra were acquired for ions exceeding 1 × 10⁴ intensity, with maximum injection times of 1000 ms for MS and 100 ms for MS/MS.

### Database search and bioinformatics analysis

Raw MS data files were processed using Proteome Discoverer software (v1.3.0.339; Thermo Fisher Scientific) and searched against the UniProt database (taxonomy: Human) using the MASCOT search engine (v2.2; Matrix Science, London, UK). Chymotrypsin was specified as the digestion enzyme, allowing for one missed cleavage. Carbamidomethylation of cysteine and dimethylation (lysine side chains) were set as fixed modifications, while methionine oxidation, N-terminal dimethylation, and pyro-glutamate formation from N-terminal glutamine (Gln → pyro-Glu) were set as variable modifications. Mass tolerances were set to 10 ppm for MS and 0.5 Da for MS/MS. A decoy database search strategy was used to estimate the false discovery rate (FDR), which was maintained below 1%. Protein identifications required at least two peptides, including at least one unique peptide per protein.

### Lactate assay

Lactate in culture medium was measured with the Lactate Assay Kit (Cell Biolabs, #MET-5012) according to the manufacturer’s instructions. To measure intracellular lactate, cells were resuspended in ice-cold PBS, sonicated, cleared by centrifugation, and lactate levels in the supernatants were quantified using a lactate assay kit. Total intracellular lactate was calculated as molar amount (concentration × volume) and normalized to cell volume. For volumetric normalization, cell diameter was determined by bead-based flow cytometric sizing using a polydisperse microsphere standard (Invitrogen, F13838; 1–15 μm). Cell volume (V) was calculated assuming spherical geometry (V = 4/3 πr³, where r is cell radius). Intracellular lactate concentrations were expressed as mM (mol per cell volume).

### Intracellular pH measurement

For bulk pHi measurements, THP-1-derived macrophages were loaded with 0.1 μM BCECF-AM in HBSS for 30 min at 37 °C, washed with phenol red–free RPMI, and incubated with or without nigericin. BCECF fluorescence was recorded at 535 nm emission with sequential excitation at 490 nm and 440 nm using a SpectraMax iD3 microplate reader (Molecular Devices). Fluorescence ratios (F490/F440) were calculated to determine intracellular pH (pHi). Intracellular pH values were obtained from calibration curves generated in parallel using high-K^+^ buffers. For calibration, cells were incubated in phenol red–free RPMI-based buffers adjusted to defined pH values and supplemented with 135 mM KCl, 10 μM nigericin, and 10 μM valinomycin. Resulting F490/F440 ratios were plotted against buffer pH to generate calibration curves for pHi determination. For single-cell pHi measurements, THP-1 cells were differentiated, BCECF-loaded, washed, and imaged after 8 min nigericin treatment. Fluorescence imaging was performed using an ImageXpress Pico system (Molecular Devices), and pHi values were estimated from calibration curves as above. Approximately 100 cells per condition were analyzed, and images were processed in ImageJ.

### Measurement of mitochondrial membrane potential

Mitochondrial membrane potential (ΔΨm) was assessed using tetramethylrhodamine methyl ester (TMRM; MitoProbe TMRM Kit, Invitrogen). Cells were incubated with 20 nM TMRM for 30 min at 37 °C and collected for flow cytometric analysis.

### Mitochondrial and cellular ROS measurements

Mitochondrial and cellular ROS were measured using MitoSOX Red and H_2_-DCFDA (Thermo Fisher Scientific), respectively. Cells were incubated with 1 μM MitoSOX Red or 0.3 μM H_2_-DCFDA at 37 °C for 30 min and analyzed by flow cytometry as described previously [10]

### Potassium efflux assay

Potassium efflux was measured using Asante Potassium Green-4 (APG-4; Interchim). Cells were incubated with 1 μM APG-4 at 37 °C for 45 min, stimulated with nigericin as indicated, and analyzed immediately by flow cytometry [10].

### Statistical analysis

Statistical analyses were conducted using SPSS version 13.0 (SPSS Inc., Chicago, IL, USA). Sample sizes were determined based on established standards in the field and prior studies using comparable experimental systems. No biological samples or animals were excluded from the analysis unless predefined technical failure occurred. Animals were randomly assigned to experimental groups using simple random allocation. For in vitro experiments, cells from the same culture were evenly distributed across conditions. The investigator was not blinded to group allocation. Survival curves were generated using the Kaplan–Meier method and compared using the log-rank test [28]. For in vitro experiments, statistical significance was assessed using the two-tailed Student’s *t*-test or one-way ANOVA followed by Tukey’s HSD or LSD post hoc test, as indicated and described previously [34]. Serum osmolarity, blood pH, and sodium concentration were analyzed as dependent physiological variables using multivariate general linear modeling, with treatment as the fixed factor. Multivariate significance was evaluated using Pillai’s Trace, followed by univariate analyses when appropriate. Data from multivariate analyses are presented as estimated marginal means with 95% confidence intervals. Unless otherwise indicated, data are presented as mean ± SD) or SEM, as specified in the figure legends. A *P* value < 0.05 was considered statistically significant.

## Results

### Intracellular lactic acid promotes NLRP3 inflammasome activation

**Fig. 1:**
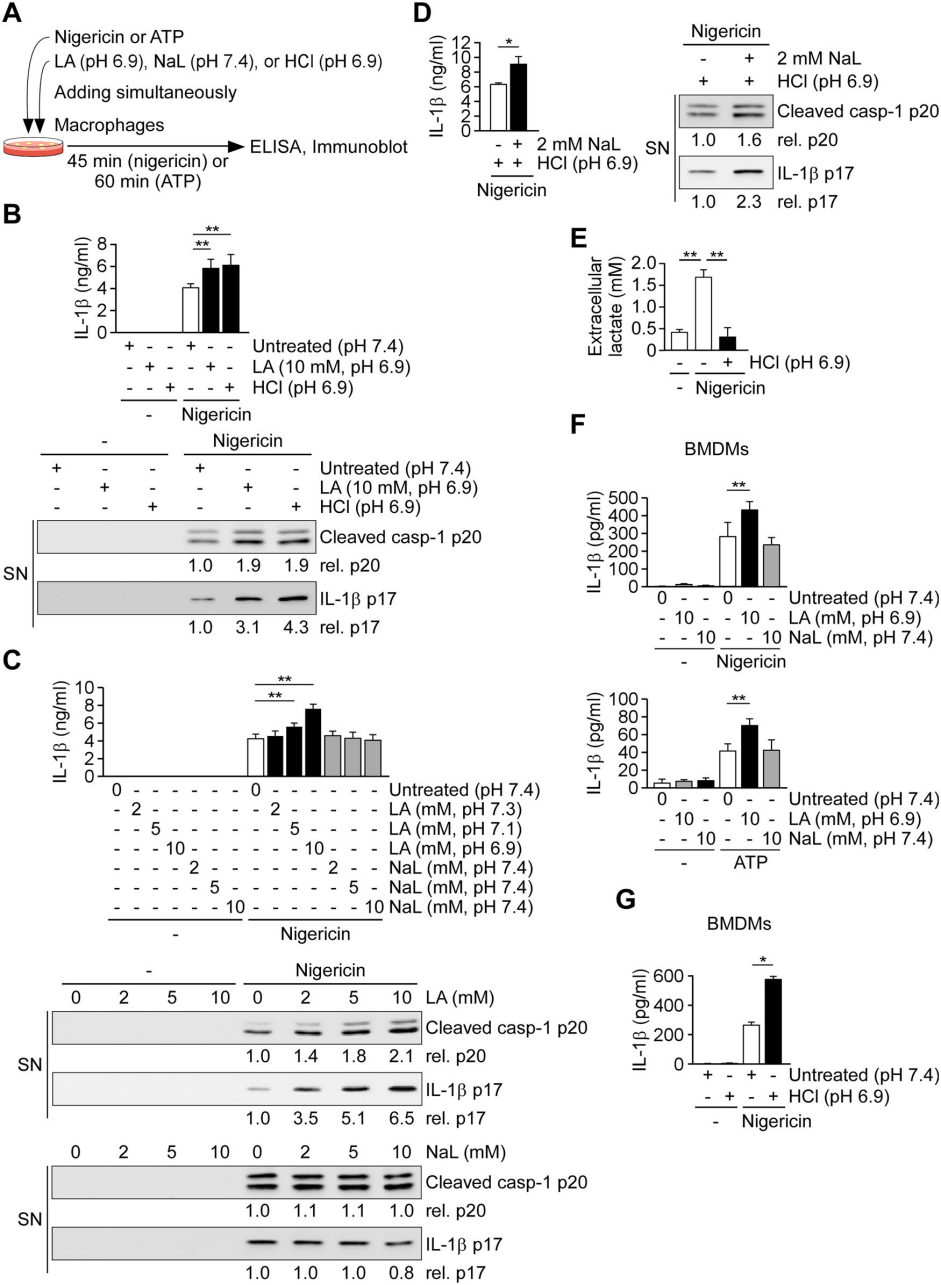
Intracellular lactic acid promotes NLRP3 inflammasome activation via intracellular acidification. **A** Schematic of the experimental design. Macrophages were stimulated with nigericin or ATP in the presence of lactic acid (LA, pH 6.9), sodium lactate (NaL, pH 7.4), or hydrochloric acid (HCl, pH 6.9). IL-1β secretion and protein expression were assessed 45 min (nigericin) or 60 min (ATP) post-treatment by ELISA and western blot (WB), respectively. **B** IL-1β concentrations (*n* = 4) and immunoblots of caspase-1 p20 and IL-1β p17 in supernatants (SN) of cells treated as indicated. The immunoblot is representative of three independent experiments. **C** Dose-dependent effects of LA and NaL on IL-1β secretion (*n* = 6) and immunoblots of caspase-1 and IL-1β. The immunoblot is representative of three independent experiments. **D** IL-1β secretion (*n* = 4) and immunoblots in cells treated with NaL and HCl as indicated in the presence of nigericin. The immunoblot is representative of three independent experiments. **E** Extracellular lactate levels measured following stimulation with nigericin ± HCl (*n* = 3). **F** IL-1β secretion from bone marrow-derived macrophages (BMDMs) treated with LA or NaL and stimulated with nigericin (top) or ATP (bottom) (*n* = 4). **G** IL-1β secretion in BMDMs treated with HCl and nigericin (*n* = 4). All data are shown as mean ± SD. *P < 0.05, **P < 0.01 by one-way ANOVA with Tukey’s HSD post hoc test.

Lactic acid fermentation is required for NLRP3 inflammasome activation in macrophages [10]; however, the underlying mechanisms remain unclear. Lactic acid, a byproduct of lactic acid fermentation, dissociates into lactate and a proton, contributing to intracellular acidification. To maintain cellular pH homeostasis, cells co-transport lactate and protons via monocarboxylate transporters (MCTs), utilizing the proton gradient between the cytoplasm and the extracellular environment [35]. To investigate the role of intracellular lactic acid in NLRP3 inflammasome activation, we increased intracellular lactic acid levels by enhancing its uptake through extracellular lactic acid treatment [36] and by reducing its excretion using extracellular hydrochloric acid (HCl) treatment at pH 6.9 (Fig. 1A) [37]. Our results showed that while neither extracellular lactic acid nor HCl alone activated the NLRP3 inflammasome, both treatments enhanced IL-1β secretion and promoted the production of mature IL-1β (p17) and active caspase-1 (p20) in THP-1-differentiated macrophages stimulated with the NLRP3 activator nigericin, compared to nigericin stimulation alone (Fig. 1B). In contrast, sodium lactate did not exert the same effect at a neutral pH of 7.4 (Fig. 1C), but did induce a similar effect under acidic conditions (pH 6.9) (Fig. 1D). In addition, we confirmed that extracellular HCl at pH 6.9 inhibited cellular lactic acid excretion. Lactic acid excretion, which was induced in THP-1-derived macrophages upon nigericin stimulation [10], was significantly reduced by HCl treatment (Fig. 1E). Consistently, extracellular lactic acid at pH 6.9, but not sodium lactate at neutral pH, enhanced IL-1β secretion in bone marrow-derived macrophages (BMDMs) stimulated with the NLRP3 activators nigericin and ATP, compared to stimulation with nigericin and ATP alone (Fig. 1F). In addition, extracellular HCl at pH 6.9 enhanced IL-1β secretion in BMDMs stimulated with nigericin (Fig. 1G). The effect of extracellular HCl on ATP-induced NLRP3 inflammasome activation was not examined, as the intrinsic acidity of the 5 mM ATP used in this study reduced the culture medium pH to approximately 6.9. We next examined whether the lactate receptor GPR81, which has been implicated in lactate-mediated immunosuppression [38], contributed to this effect. Pharmacological modulation of GPR81 did not alter extracellular lactic acid–mediated enhancement of NLRP3 inflammasome activation. Neither the GPR81 antagonist 3-OBA nor the agonist 3Cl-5OH-BA affected IL-1β secretion in nigericin-stimulated THP-1–derived macrophages in the presence or absence of extracellular lactic acid (Supplementary Fig. S1), indicating that this effect is independent of GPR81 signaling. Together, these results suggest that lactic acid in the cytoplasm contributes to the activation of the NLRP3 inflammasome in macrophages.

### Endogenous lactic acid production is required for NLRP3 inflammasome assembly and activation

**Fig. 2:**
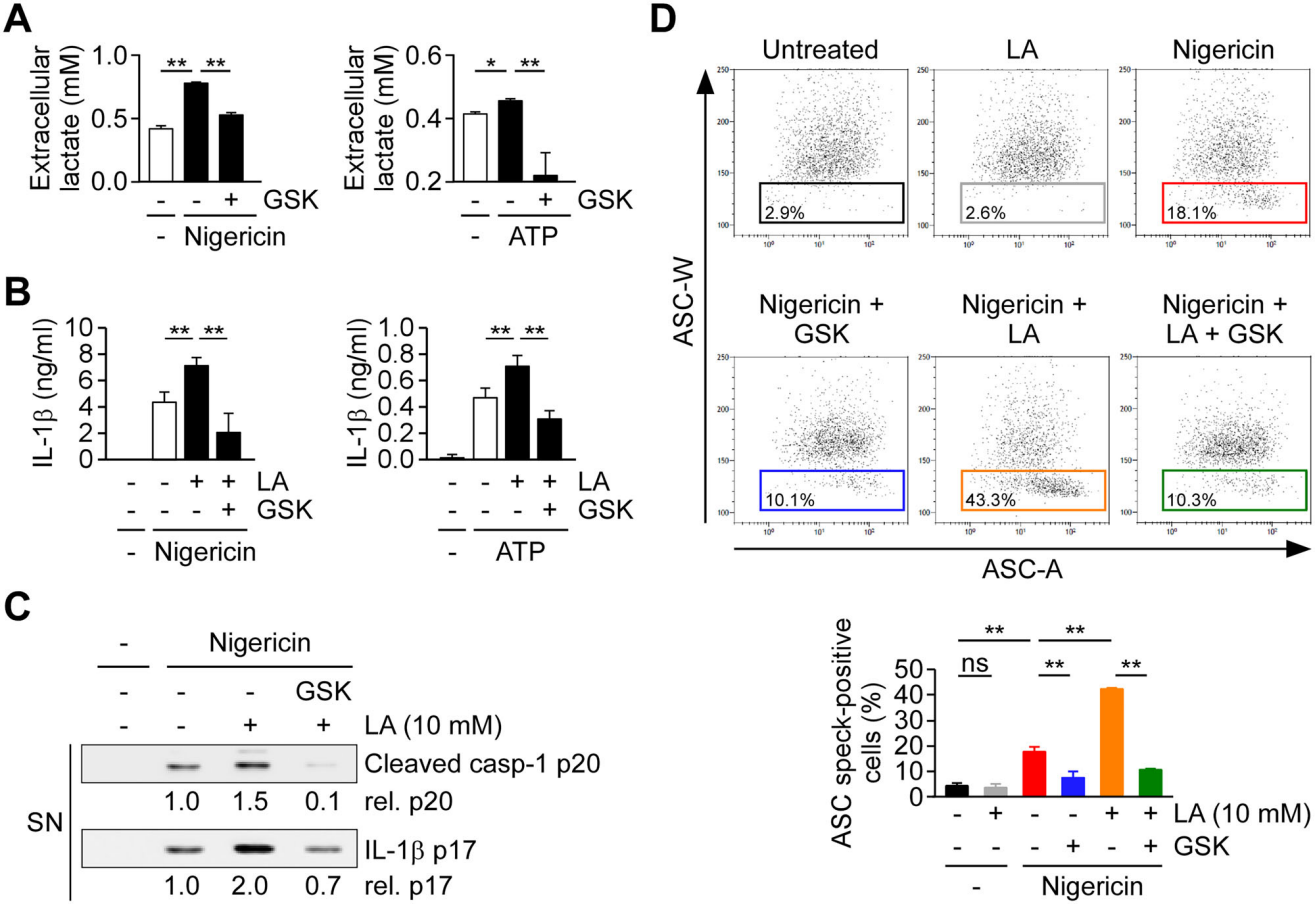
Endogenous lactic acid production is required for NLRP3 inflammasome assembly. **A** Extracellular lactate concentrations in THP-1-derived macrophages stimulated with nigericin (*n* = 5) or ATP (*n* = 3) and treated with or without the lactate dehydrogenase inhibitor GSK2837808A (GSK). *P < 0.05, **P < 0.01, determined by one-way ANOVA with LSD post hoc test. **B** IL-1β secretion measured by ELISA in cells stimulated with nigericin or ATP and treated with 10 mM extracellular LA ± GSK (*n* = 6). **C** Immunoblots of caspase-1 p20 and IL-1β p17 in supernatants from cells treated as in (**B**). Relative band intensities are shown below each blot. The immunoblot is representative of three independent experiments. **D** Flow cytometric analysis of ASC speck formation in ASC–mCherry-expressing THP-1 macrophages treated with nigericin and/or LA, with or without GSK. Representative plots (top) and quantification of ASC speck–positive cells (bottom); speck-positive cells were defined by reduced ASC-W:ASC-A signal profiles (boxed regions) (*n* = 4). *P < 0.05, **P < 0.01, ns, not significant by one-way ANOVA with Tukey’s HSD post hoc test. All data are shown as mean ± SD.

Because extracellular lactic acid and HCl alone were insufficient to activate the NLRP3 inflammasome, their presence promoted NLRP3 inflammasome activation only when a NLRP3 activator was present (Fig. 1B). In addition, glycolysis is activated upon NLRP3 inflammasome activation in macrophages, leading to subsequent lactic acid production [10]. Therefore, we investigated the role of endogenous lactic acid production in NLRP3 inflammasome activation by examining the effects of the lactate dehydrogenase inhibitor GSK2837808A on IL-1β secretion, caspase-1 activation, and NLRP3 inflammasome complex formation in THP-1-derived macrophages treated with extracellular lactic acid. Stimulation with nigericin or ATP increased extracellular lactate levels, an effect abolished by GSK2837808A treatment, indicating that lactic acid production is LDH- and glycolysis-dependent (Fig. 2A). Co-treatment with extracellular lactic acid enhanced IL-1β secretion in nigericin- or ATP-stimulated cells, an effect that was suppressed by GSK2837808A (Fig. 2B). Similarly, GSK2837808A reduced the expression of mature IL-1β (p17) and active caspase-1 (p20) in macrophages co-treated with nigericin and lactic acid (Fig. 2C). During NLRP3 inflammasome assembly, ASC molecules oligomerize to form specks that recruit procaspase-1 prior to its activation. To assess inflammasome complex formation, we performed time-of-flight inflammasome evaluation analysis to quantify ASC speck-positive and speck-negative macrophages, as previously described [31]. In ASC-mCherry-expressing THP-1 cells, nigericin treatment increased the percentage of ASC speck-containing cells from 4.3% at baseline to 17.9% (Fig. 2D). Co-treatment with lactic acid further elevated this percentage to 42.9%, whereas inhibition of lactic acid production with GSK2837808A reduced it to 10.7%. These findings indicate that endogenous lactic acid production is required for optimal NLRP3 inflammasome assembly and activation. We next investigated whether endogenous lactic acid production also regulates other inflammasome platforms. Transfection with poly(dA:dT) activated the AIM2 inflammasome, as evidenced by the generation of caspase-1 p20 and mature IL-1β p17, as well as increased IL-1β secretion. Notably, all of these responses were markedly suppressed by GSK2837808A treatment (Supplementary Fig. S2A,B). Together, these findings indicate that endogenous lactic acid production broadly supports inflammasome activation, extending beyond NLRP3 to include the AIM2 inflammasome.

### Intracellular lactic acidification promotes NLRP3 inflammasome activation

**Fig. 3:**
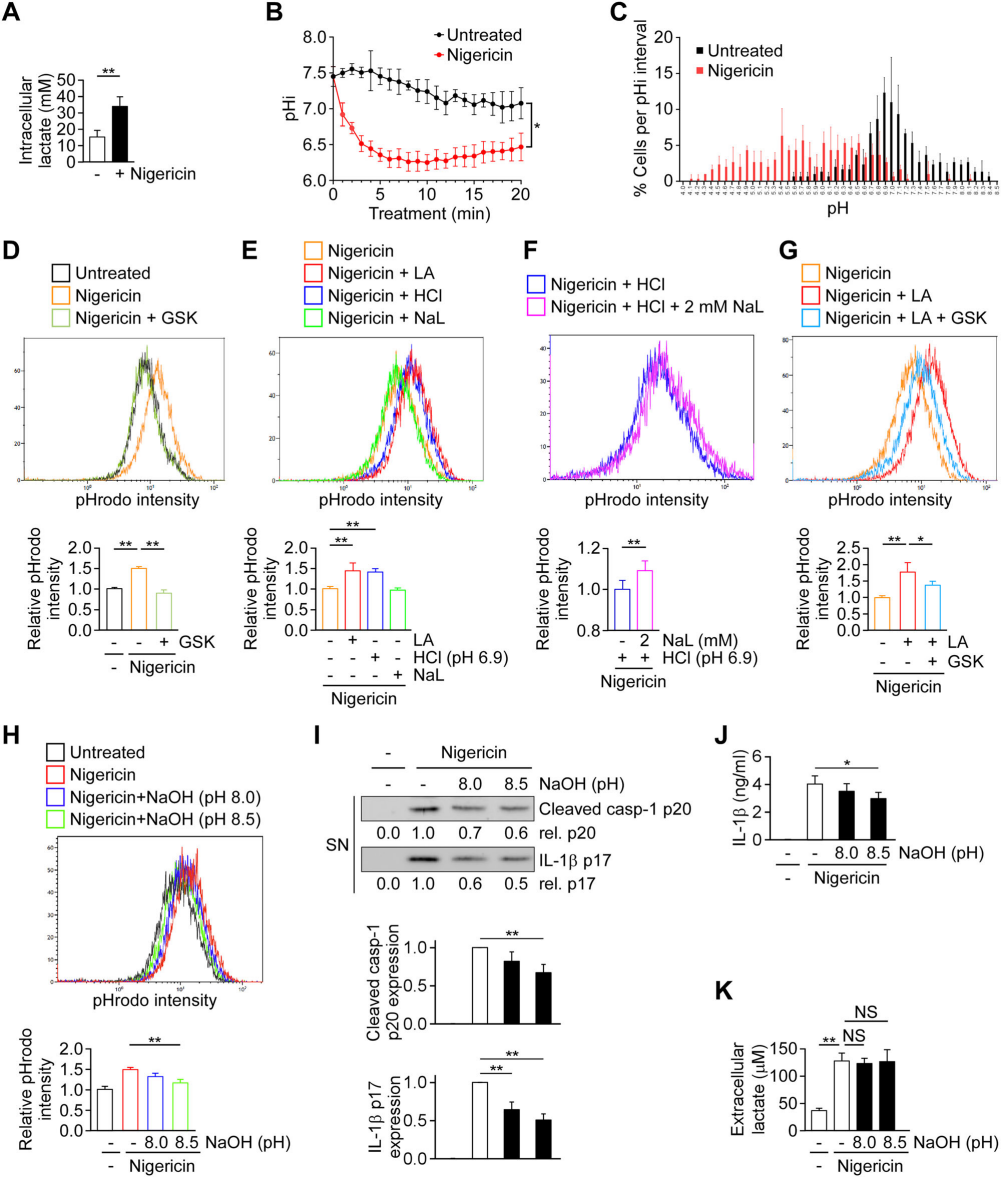
Intracellular lactic acidification promotes NLRP3 inflammasome activation. **A** Intracellular lactate concentrations in THP-1–derived macrophages left untreated or stimulated with nigericin (*n* = 4). **B** Time-course analysis of mean intracellular pH (pHi) measured in live cells following nigericin stimulation (*n* = 3). **C** Single-cell pH distribution analysis (*n* = 3). **D**–**H** Flow cytometric analysis of intracellular pH using pHrodo staining in THP-1-derived macrophages. **D** pHrodo signal intensity in cells treated with nigericin ± GSK (*n* = 3). **E** pHrodo intensity in cells co-treated with nigericin and LA, HCl, or NaL (*n* = 4). **F** Effect of NaL co-treatment on HCl-induced intracellular acidification in nigericin-stimulated cells (*n* = 4). **G** GSK abolished lactic acid–induced acidification in nigericin-stimulated cells (*n* = 4). **H** pHrodo intensity in cells treated with NaOH (pH 8.0 or 8.5) during nigericin stimulation (*n* = 3). **I** Immunoblots and quantification of caspase-1 p20 and IL-1β p17 in THP-1-derived macrophages treated with nigericin and increasing extracellular pH (*n* = 4). The immunoblot is representative of four independent experiments. **J** IL-1β secretion by ELISA in THP-1-derived macrophages treated with nigericin ± NaOH. **K** Extracellular lactate levels measured in THP-1-derived macrophages treated with nigericin ± NaOH (*n* = 4). All data are shown as mean ± SD. *P < 0.05, **P < 0.01, NS, not significant by one-way ANOVA with Tukey’s HSD post hoc test.

Lactic acid dissociates into lactate and a proton, potentially leading to intracellular acidification. To assess whether intracellular lactic acid and pH levels were affected during NLRP3 inflammasome activation, we quantified intracellular lactate concentrations and measured cytosolic pH in intact macrophages. Stimulation of THP-1-derived macrophages with nigericin induced a marked accumulation of intracellular lactate, reaching 33.8 ± 5.5 mM compared with 15.0 ± 3.5 mM in untreated cells (Fig. 3A), consistent with robust glycolysis-dependent lactate production during inflammasome activation. Concomitantly, live-cell imaging revealed a rapid and sustained decrease in mean intracellular pH from 7.3 ± 0.1 to 6.3 ± 0.1 within 8 min of nigericin treatment (Fig. 3B). Single-cell pH distribution analysis further revealed pronounced heterogeneity within the macrophage population, with a distinct subset of cells undergoing profound cytosolic acidification, reaching pH values as low as 4.1 ± 0.1 (Fig. 3C). Consistent with these findings, nigericin markedly increased the fluorescence of the pH-sensitive indicator pHrodo, reflecting intracellular acidification in THP-1–derived macrophages (Fig. 3D). This increase was abolished by inhibition of lactate dehydrogenase with GSK2837808A, demonstrating that endogenous lactic acid production is required for nigericin-induced intracellular acidification. Furthermore, extracellular lactic acid and HCl promoted NLRP3 inflammasome activation (Fig. 1B) as well as intracellular acidification in macrophages stimulated with nigericin, whereas sodium lactate did not (Fig. 3E). Although sodium lactate alone did not promote intracellular acidification, it was able to do so under acidic conditions (pH 6.9) (Fig. 3F). Importantly, the promotion of intracellular acidification by extracellular lactic acid was abolished by GSK2837808A treatment, demonstrating that this effect depended on endogenous lactic acid production (Fig. 3G). Together, these results indicate that lactic acid production induced by NLRP3 activators contributes to intracellular acidification in macrophages. Next, we examined the role of intracellular acidification in NLRP3 inflammasome activation. Given that extracellular alkalization has been reported to increase intracellular pH [39], we investigated the effect of extracellular alkaline pH 8.0 and 8.5 (adjusted with NaOH) on NLRP3 inflammasome activation. We found that extracellular pH 8.5 protected cells from nigericin-induced intracellular acidification (Fig. 3H), and concurrently inhibited caspase-1 activation, IL-1β maturation (Fig. 3I), and IL-1β secretion (Fig. 3J), without affecting lactate excretion (Fig. 3K).

### Intracellular lactic acidification licenses NLRP3 inflammasome activation through parallel ROS- and PKR-dependent pathways

**Fig. 4:**
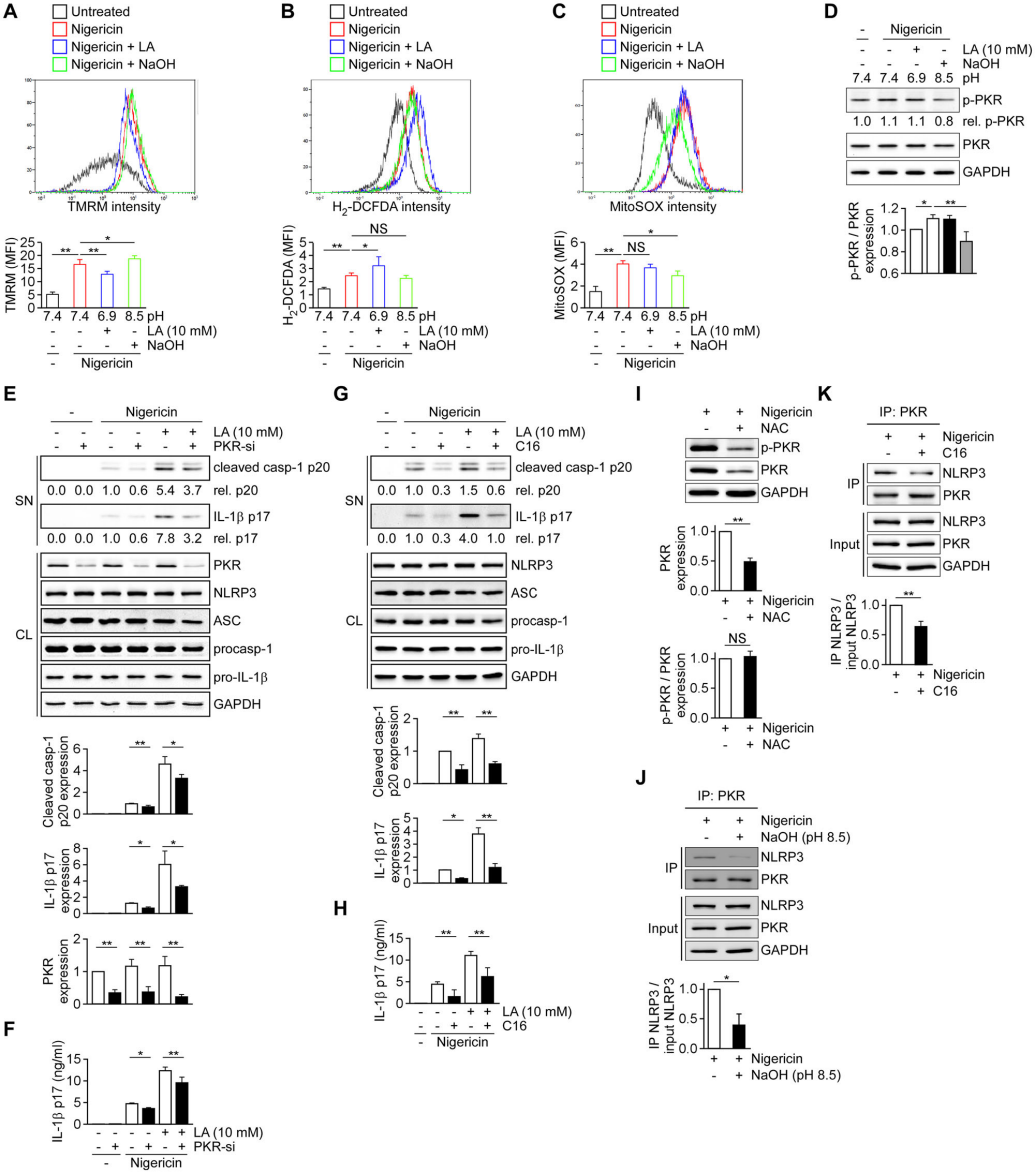
Intracellular lactic acidification promotes NLRP3 inflammasome activation by reshaping mitochondrial redox signaling and engaging PKR-dependent pathways. **A**–**C** THP-1–derived macrophages were stimulated with nigericin in the presence or absence of extracellular lactic acid (LA, 10 mM; pH 6.9) or extracellular alkalinization (NaOH; pH 8.5). Mitochondrial membrane potential (ΔΨm) (**A**) total cellular reactive oxygen species (ROS) (**B**) and mitochondrial ROS (**C**) were assessed by TMRM (*n* = 4), H₂-DCFDA (*n* = 6), and MitoSOX (*n* = 4) staining, respectively, followed by flow cytometric analysis. Representative histograms (top) and quantification of mean fluorescence intensity (MFI) (bottom) are shown. **D** Immunoblot analysis of phosphorylated PKR (p-PKR) and total PKR under the indicated conditions (*n* = 4). **E**, **F** Immunoblot analysis of cleaved caspase-1 (p20) and mature IL-1β (p17) in culture supernatants (*n* = 3), inflammasome components in cell lysates (*n* = 4), and corresponding ELISA quantification (*n* = 6) in control or PKR-silenced cells. **G**, **H** Immunoblot analysis of cleaved caspase-1 (p20) and mature IL-1β (p17) in culture supernatants (*n* = 3), inflammasome components in cell lysates (*n* = 4), and corresponding ELISA quantification (*n* = 6) in cells treated with the PKR kinase inhibitor C16. **I** Immunoblot analysis of PKR and phosphorylated PKR (p-PKR) in nigericin-stimulated cells treated with the ROS scavenger N-acetylcysteine (NAC) (*n* = 3). **J**, **K** Co-immunoprecipitation analysis of PKR–NLRP3 interaction in nigericin-stimulated cells treated with extracellular alkalinization (**J**) or the PKR inhibitor C16 (**K**) (*n* = 3 each). All data are presented as mean ± SD. Statistical significance was determined by one-way ANOVA with Tukey’s HSD post hoc test or Student’s *t*-test, as appropriate. *P* < 0.05; *P* < 0.01; NS not significant.

Given that intracellular lactic acidification was tightly coupled to NLRP3 inflammasome activation, we next investigated how it modulates upstream stress signals and signaling intermediates that license inflammasome assembly. Nigericin is a K⁺/H⁺ ionophore that activates the NLRP3 inflammasome through induction of potassium efflux [9]. Consistent with previous reports, nigericin reduced intracellular potassium levels; however, this effect was not altered by extracellular lactic acid or extracellular alkalinization to pH 8.5 (Supplementary Fig. S3), indicating that intracellular lactic acidification does not regulate NLRP3 activation by modulating potassium efflux. Beyond ion flux, the K⁺/H⁺ ionophore activity of nigericin has been shown to lower intramitochondrial pH and ΔpH, leading to a compensatory increase in mitochondrial membrane potential (ΔΨm) and augmented reactive oxygen species (ROS) production [40]. Consistent with this mechanism, nigericin stimulation markedly increased ΔΨm, as assessed by TMRM staining (Fig. 4A), total cellular ROS production measured by H₂-DCFDA fluorescence (Fig. 4B), and mitochondrial ROS production assessed by MitoSOX staining (Fig. 4C). Notably, extracellular lactic acid enhanced intracellular acidification (Fig. 3E) and concomitantly promoted mitochondrial depolarization (Fig. 4A) together with increased total cellular ROS production (Fig. 4B). In contrast, extracellular alkalinization (pH 8.5) protected cells from nigericin-induced intracellular acidification (Fig. 3H) while further increasing mitochondrial membrane potential (Fig. 4A) and suppressing mitochondrial ROS production (Fig. 4C). Together, these data indicate that intracellular acidification reshapes mitochondrial bioenergetic and promotes ROS production during NLRP3 activation.

Because blocking intracellular lactic acidification with GSK2837808A was previously shown to suppress PKR phosphorylation [10], a process implicated in regulating NLRP3 inflammasome activation via physical interaction between PKR and NLRP3 [14], we next assessed PKR activation under these conditions. Nigericin induced PKR phosphorylation, which was significantly attenuated by extracellular alkalinization (Fig. 4D). Moreover, genetic silencing of PKR or pharmacological inhibition of PKR kinase activity with C16 abolished both nigericin-induced and extracellular lactic acid–mediated enhancement of caspase-1 cleavage and IL-1β maturation (Fig. 4E–H), demonstrating that PKR activity is required for intracellular lactic acidification–driven inflammasome activation. To further delineate the relationship between ROS signaling and PKR activation, we treated nigericin-stimulated cells with the ROS scavenger N-acetylcysteine (NAC). NAC treatment markedly reduced total PKR protein expression but did not affect PKR phosphorylation levels relative to PKR abundance (Fig. 4I), indicating that ROS regulates PKR expression rather than its phosphorylation status. These findings suggest that ROS generation and PKR phosphorylation represent mechanistically distinct signaling axes downstream of intracellular lactic acidification. To assess whether intracellular lactic acidification influences PKR–NLRP3 interaction, we conducted co-immunoprecipitation assays using PKR as bait. THP-1–derived macrophages stimulated with nigericin, with or without extracellular pH 8.5 treatment, were lysed and subjected to PKR pull-down. NLRP3 levels in PKR immunoprecipitates were markedly reduced in cells treated with extracellular pH 8.5 compared to controls (Fig. 4J). Similarly, inhibition of PKR kinase activity disrupted PKR–NLRP3 interaction (Fig. 4K). Collectively, these data support a model in which intracellular lactic acidification promotes NLRP3 inflammasome activation through two parallel pathways: one involving mitochondrial ROS production, and another involving PKR phosphorylation that facilitates PKR–NLRP3 interaction and inflammasome assembly.

### Lactic acid induces mature-like IL-1β independently of the NLRP3 inflammasome

**Fig. 5:**
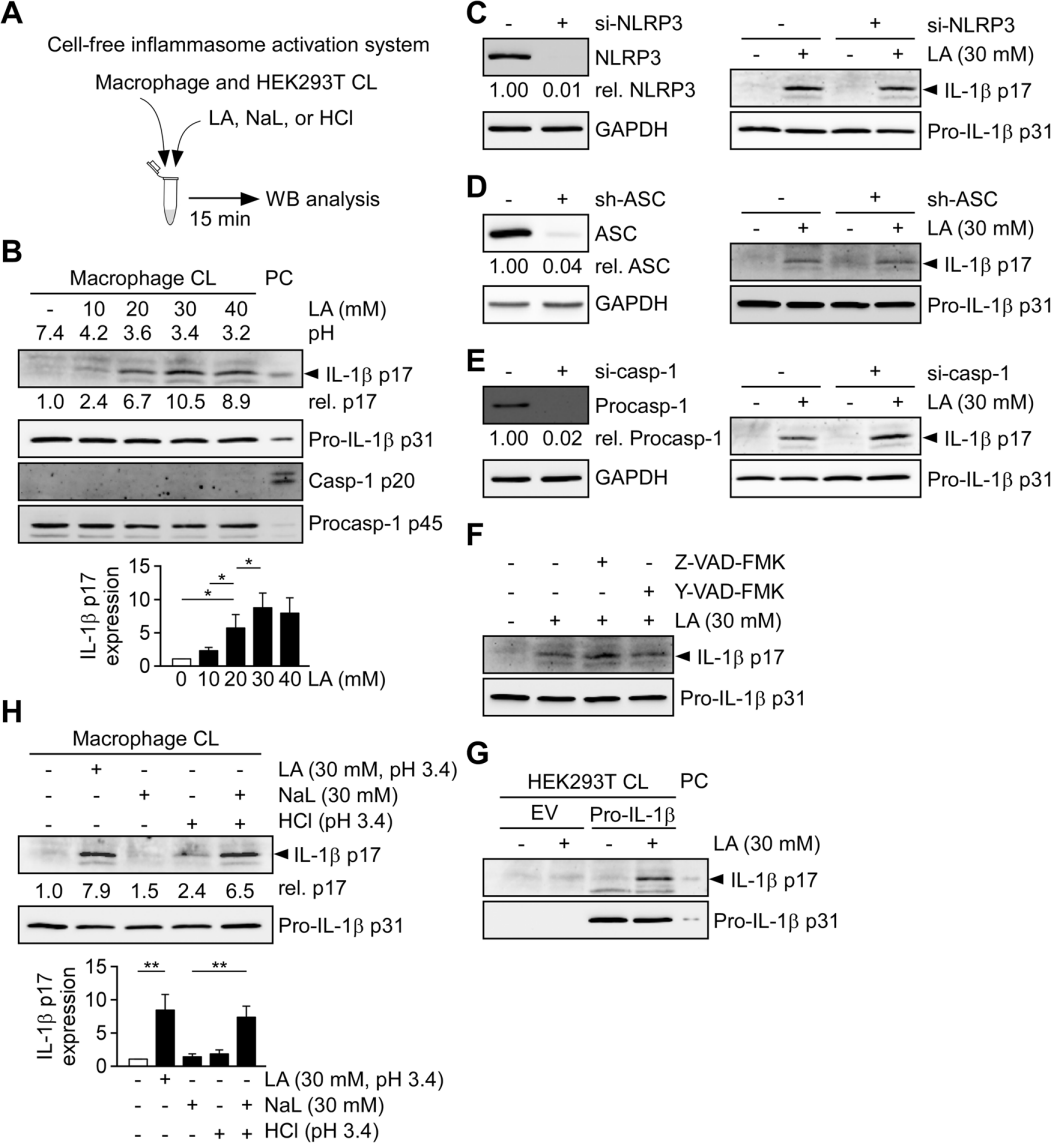
Lactic acid induces pro-IL-1β cleavage independently of the NLRP3 inflammasome. **A** Schematic of the cell-free NLRP3 inflammasome activation assay. Whole-cell lysates (CL) from THP-1-derived macrophages or HEK293T cells were incubated with LA, NaL, or HCl, followed by immunoblot analysis. **B** Dose-dependent effects of LA on IL-1β cleavage in macrophage lysates. Immunoblots show IL-1β, pro-IL-1β, caspase-1, and procaspase-1. Densitometric quantification of IL-1β relative to pro-IL-1β is shown (bottom, *n* = 6). PC, positive control (nigericin-treated lysates). Immunoblots of IL-1β in lysates from cells with siRNA knockdown of NLRP3 (**C**) ASC (**D**) or caspase-1 (**E**) followed by LA treatment. GAPDH and pro-IL-1β serve as loading controls. The immunoblot is representative of three independent experiments. **F** Immunoblots of IL-1β in macrophage lysates treated with LA in the presence of the pan-caspase inhibitor Z-VAD-FMK or the caspase-1–specific inhibitor Y-VAD-FMK. The immunoblot is representative of three independent experiments. **G** Immunoblots of IL-1β in HEK293T lysates transfected with pro-IL-1β or empty vector (EV), followed by LA treatment. The immunoblot is representative of three independent experiments. **H** Immunoblot of IL-1β and pro-IL-1β in macrophage lysates treated with LA. Densitometric quantification of IL-1β relative to pro-IL-1β is shown (bottom, *n* = 3). All data are shown as mean ± SD. *P < 0.05, **P < 0.01, NS, not significant by one-way ANOVA with Tukey’s HSD post hoc test.

To investigate how intracellular lactic acidification activates the NLRP3 inflammasome, we established a cell-free inflammasome activation system to assess the direct effect of lactic acid on the cleavage of procaspase-1 and pro-IL-1β. Whole protein lysates were prepared using RIPA buffer from THP-1-derived macrophages or HEK293T cells without prior NLRP3 stimulation (Fig. 5A). Lactic acid induced the cleavage of pro-IL-1β into a mature-like form in a dose-dependent manner (10–30 mM; Fig. 5B). Notably, the molecular weight of the cleaved IL-1β was comparable to that of mature IL-1β generated by nigericin stimulation in THP-1-derived macrophages (Fig. 5B). However, active caspase-1 was not detected following lactic acid treatment (Fig. 5B), suggesting a caspase-independent cleavage mechanism. To further assess whether canonical inflammasome components were involved, we silenced NLRP3 (Fig. 5C), ASC (Fig. 5D), or caspase-1 (Fig. 5E) using specific siRNAs or shRNA. Additionally, caspase activity was inhibited using the pan-caspase inhibitor Z-VAD-FMK or the caspase-1-specific inhibitor Y-VAD-FMK (Fig. 5F). None of these interventions affected lactic acid–induced IL-1β cleavage. To confirm that this process was independent of the canonical NLRP3 inflammasome, we repeated the experiments using HEK293T cells, which lack endogenous inflammasome components [41]. Consistently, lactic acid induced pro-IL-1β cleavage in lysates from HEK293T cells transfected with pro-IL-1β (Fig. 5G). To delineate the mechanism, we compared the effects of lactic acid, sodium lactate, and HCl. Only lactic acid induced IL-1β cleavage (Fig. 5H). However, when the lysate pH was adjusted to 3.4 using HCl—mimicking the acidification induced by 30 mM lactic acid—sodium lactate also induced cleavage of pro-IL-1β, suggesting that lactic acid itself, rather than acidification alone, was responsible for this effect. Collectively, these results demonstrate that lactic acid, rather than low pH alone, can induce pro-IL-1β cleavage into a mature-like form through an NLRP3 inflammasome-independent mechanism.

### Lactic acid mimics caspase-1 via a carboxyl group-dependent mechanism

**Fig. 6:**
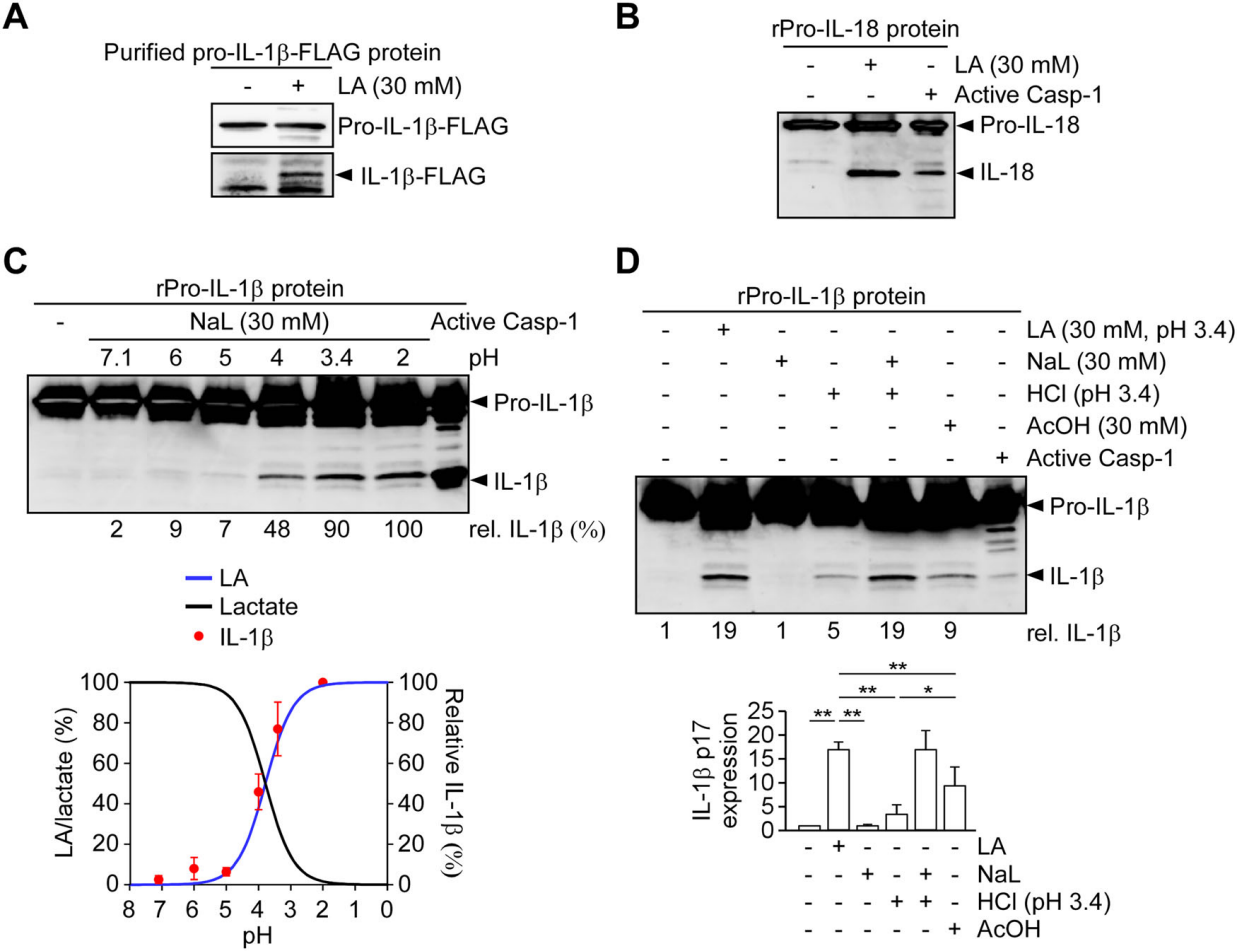
Lactic acid directly cleaves pro-inflammatory cytokines via a carboxyl group– and pH-dependent mechanism. **A** Immunoblot of IL-1β–FLAG generated from immunoprecipitated pro-IL-1β–FLAG protein using anti-FLAG antibody from HEK293T cells transfected with pro-IL-1β–FLAG, followed by treatment with LA. The immunoblot is representative of three independent experiments. **B** Immunoblot of IL-18 generated from recombinant pro-IL-18 protein treated with LA or active caspase-1 (positive control). The immunoblot is representative of three independent experiments. **C** Recombinant pro-IL-1β protein was incubated with NaL across a pH gradient or with active caspase-1. Top: immunoblot showing pH-dependent cleavage of pro-IL-1β into IL-1β. Bottom: graph showing correlation between pH-dependent conversion of lactate to LA (calculated using the Henderson–Hasselbalch equation) and IL-1β generation (*n* = 3). **D** Recombinant pro-IL-1β was treated with LA, NaL, HCl, or acetic acid (AcOH). Top: immunoblot showing IL-1β p17 production; bottom: densitometric quantification of IL-1β p17 expression (*n* = 4). All data are shown as mean ± SD. *P < 0.05, **P < 0.01, NS, not significant by one-way ANOVA with Tukey’s HSD post hoc test.

To determine whether lactic acid directly cleaves pro-IL-1β to generate mature-like IL-1β, we used immunoprecipitated pro-IL-1β protein prepared from HEK293T cells transiently transfected with pro-IL-1β. Our results showed that lactic acid was able to cleave the immunoprecipitated pro-IL-1β and generate a mature-like IL-1β protein (Fig. 6A). We next examined whether lactic acid could also mediate the cleavage of pro-IL-18, another caspase-1 substrate regulated by the NLRP3 inflammasome [39]. Similarly, lactic acid induced the cleavage of recombinant pro-IL-18 protein, producing a mature-like IL-18 fragment of approximately 18 kDa, comparable to the product generated by active caspase-1 (Fig. 6B). These findings suggest that lactic acid exhibits a broader cleavage activity toward caspase-1 substrates. Since lactate and lactic acid are interconvertible depending on solution pH, we further evaluated the pH dependency of lactic acid-induced IL-1β cleavage. Lactic acid, but not sodium lactate, induced a mature-like IL-1β expression in a pH-dependent manner, similar to the cleavage observed with active caspase-1 (Fig. 6C, top panel). Notably, the percentage of mature-like IL-1β expression correlated with the conversion rate of lactate to lactic acid under different pH conditions (Fig. 6C, bottom panel). These results demonstrate that lactic acid, rather than lactate or acidification alone, is sufficient to directly cleave pro-IL-1β into mature-like IL-1β. Given that lactic acid—but not lactate or HCl—induced the production of mature-like IL-1β, we hypothesized that the carboxyl group of lactic acid contributes to its cleavage activity. To test this, we compared the effects of lactic acid and acetic acid, two structurally similar organic acids that both contain a carboxyl group, with lactic acid possessing a three-carbon backbone and acetic acid a two-carbon backbone. Our results showed that both lactic acid and acetic acid induced higher levels of mature-like IL-1β compared to HCl (Fig. 6D). Notably, lactic acid induced greater mature-like IL-1β expression than acetic acid (Fig. 6D). These findings support the idea that the carboxyl group of organic acids contributes to pro-IL-1β cleavage into a mature-like form, with lactic acid exhibiting stronger activity than acetic acid.

### Lactic acid cleaves pro-IL-1β at the canonical caspase-1 site Asp116

**Fig. 7:**
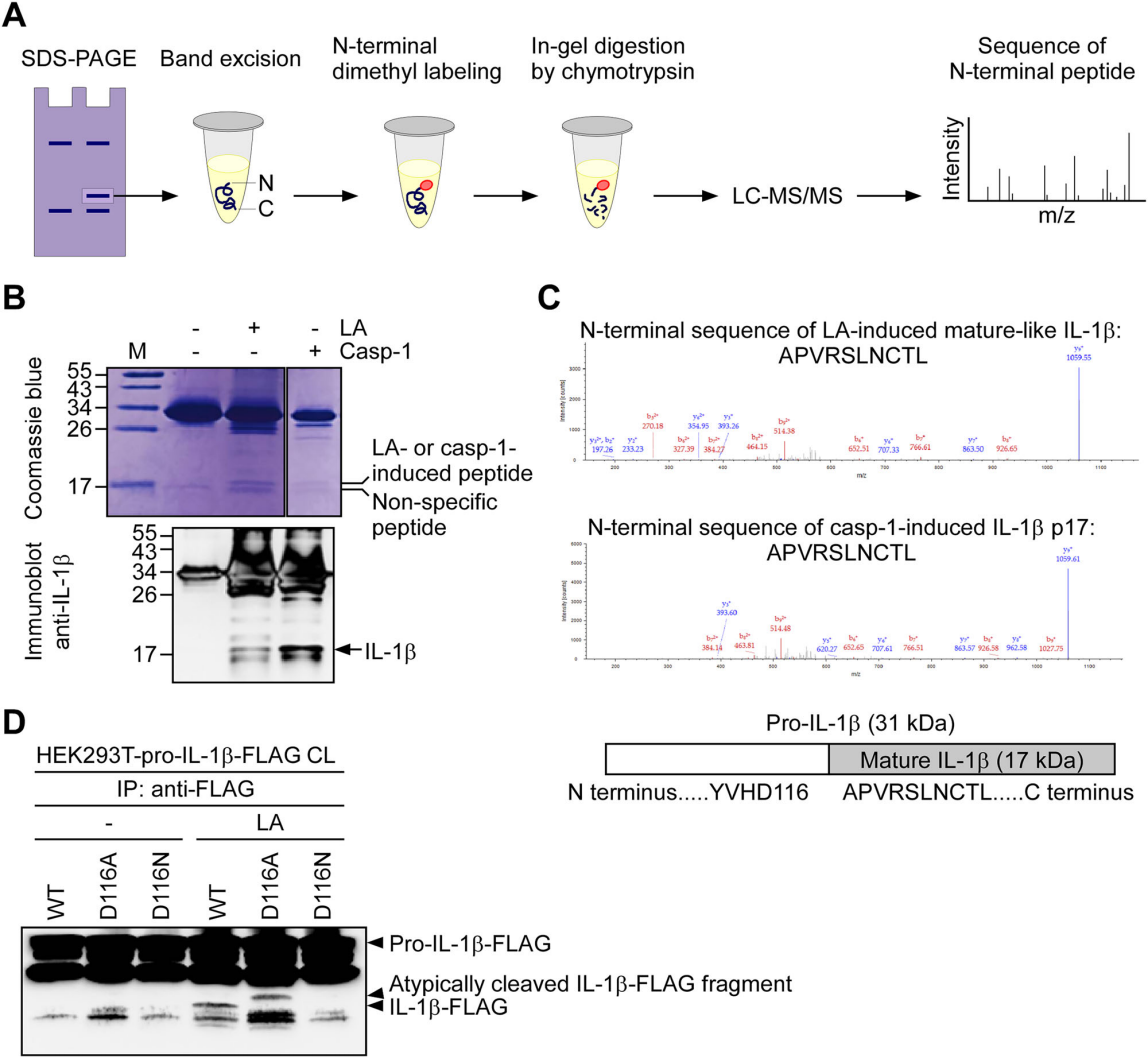
Lactic acid cleaves pro-IL-1β at the canonical caspase-1 site Asp116. **A** Schematic of the workflow for identifying the N-terminal sequence of mature-like IL-1β using dimethyl labeling and LC-MS/MS following SDS-PAGE, in-gel digestion, and chymotrypsin cleavage. **B** Coomassie staining (top) and immunoblotting (bottom) of IL-1β in samples treated with LA or active caspase-1. Bands corresponding to cleaved IL-1β were excised for mass spectrometry analysis. The immunoblot is representative of three independent experiments. **C** Representative MS/MS spectra showing the identical N-terminal sequence (APVRSLNCTL) of IL-1β generated by LA or caspase-1 cleavage. Schematic (bottom) shows the cleavage site at Asp116 in the context of full-length IL-1β. **D** Immunoblot of IL-1β-FLAG cleavage in immunoprecipitated lysates from 293T cells expressing wild-type (WT) or Asp116 mutants (D116A, D116N) of pro-IL-1β-FLAG, treated with LA. The immunoblot is representative of three independent experiments.

Although the molecular weight of lactic acid–induced mature-like IL-1β was comparable to that of IL-1β p17 generated by caspase-1, the precise identity of the lactic acid–induced mature-like IL-1β remained to be determined. To address this, we analyzed the N-terminal amino acid sequence of lactic acid–induced mature-like IL-1β using N-terminal dimethyl labeling combined with proteomic mass spectrometry (Fig. 7A). IL-1β proteins of approximately 17 kDa, generated by lactic acid or caspase-1 treatment, were separated by SDS-PAGE and excised from the gel for further analysis (Fig. 7B). The proteins were then subjected to N-terminal dimethylation and digested with chymotrypsin, followed by LC-MS/MS analysis. Strikingly, the N-terminal sequence of the lactic acid–induced mature-like IL-1β was identified as APVRSLNCTL (Fig. 7C, top panel), which was identical to the N-terminal sequence of caspase-1–generated IL-1β p17 detected in our experiment (Fig. 7C, middle panel) and consistent with previously reported caspase-1 cleavage sites (Fig. 7C, bottom panel) [42]. Sequence alignment of APVRSLNCTL with the full-length pro-IL-1β revealed that the cleavage occurred immediately downstream of Asp116, a critical residue for caspase-1–mediated processing [43]. To directly test whether Asp116 is also essential for lactic acid–induced cleavage, we generated point mutants of pro-IL-1β in which Asp116 was substituted with either alanine or asparagine. Our results showed that both mutations abolished IL-1β p17 production in response to lactic acid, while wild-type pro-IL-1β was effectively cleaved (Fig. 7D). These findings indicate that lactic acid, like caspase-1, cleaves pro-IL-1β at Asp116 to generate the mature IL-1β form.

### Elevated blood lactate promotes sepsis mortality via NLRP3 inflammasome activation

**Fig. 8:**
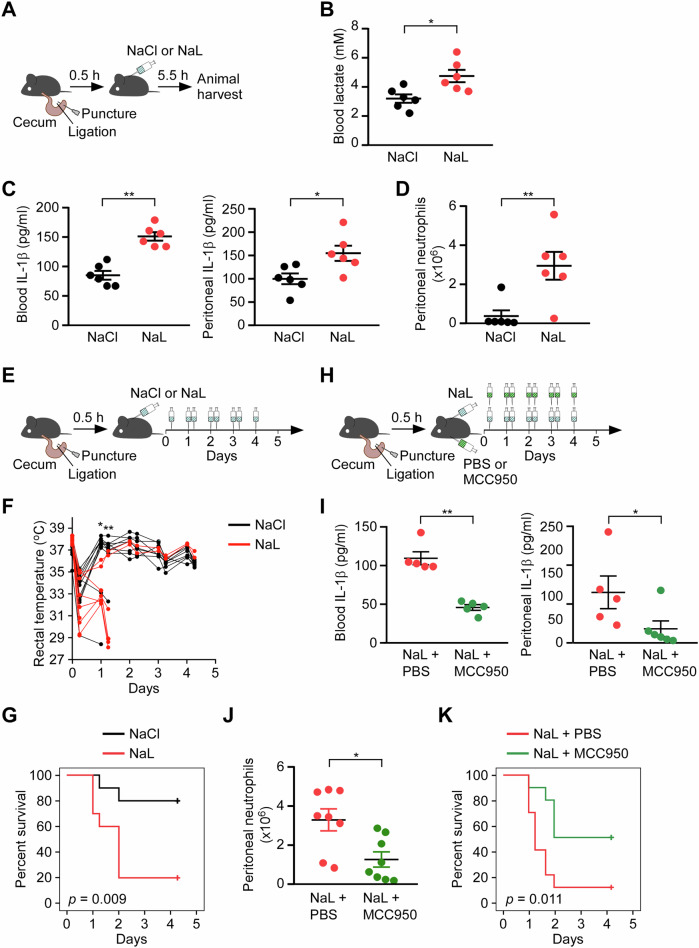
Elevated blood lactate exacerbates inflammation and mortality in a murine model of sepsis. **A** Schematic of experimental design for acute-phase analysis. Mice underwent cecal ligation and puncture (CLP) and received intraperitoneal injection of NaCl or NaL 0.5 h post-surgery; samples were harvested 5.5 h later. **B** Blood lactate concentrations (*n* = 6 per group). **C** IL-1β levels in blood (left) and peritoneal lavage fluid (right) measured by ELISA (*n* = 6 per group). **D** Total neutrophil counts in peritoneal lavage fluid (*n* = 6 per group). **E** Schematic of survival study design. CLP-treated mice received NaCl or NaL twice daily for 4 consecutive days. **F** Rectal temperature measurements over 5 days post-CLP (*n* = 10 per group). **G** Kaplan–Meier survival analysis (*n* = 10 per group). **H** Experimental design illustrating co-administration of NaL with PBS or the selective NLRP3 inhibitor MCC950 following CLP. **I** IL-1β levels in blood (left) and peritoneal fluid (right) from NaL-treated CLP mice receiving PBS or MCC950 (*n* = 5 per group). **J** Quantification of peritoneal neutrophil infiltration in NaL-treated CLP mice administered PBS or MCC950 (*n* = 8 per group). **K** Kaplan–Meier survival curves of NaL-treated CLP mice with or without MCC950 treatment (*n* = 10 per group). All data are presented as mean ± SEM of biologically independent animals. *P < 0.05, **P < 0.01 (Student’s *t*-test for panels **B**–**D**, **F**, **I** and **J**; log-rank test for panels **G** and **K**).

Elevated blood lactate levels and lactic acidosis are commonly observed in severe infectious diseases and are associated with increased mortality in patients with septic shock [44, 45]. In our in vitro studies using macrophages, both extracellular lactic acidification (Fig. 1B) and the combination of extracellular lactate with low pH (Fig. 1D) promoted NLRP3 inflammasome activation. To determine whether blood lactate contributes to septic shock via NLRP3 inflammasome activation in vivo, we employed the cecal ligation and puncture (CLP) model, a widely used method for inducing polymicrobial sepsis [46]. Previous studies have shown that NLRP3 inflammasome activation exacerbates mortality in CLP-treated mice [7]. We first confirmed that sodium lactate administration increased blood lactate levels in CLP mice compared to sodium chloride (NaCl) controls (Fig. 8A, B). Sodium lactate treatment also enhanced inflammatory responses, as evidenced by elevated IL-1β levels in both blood and peritoneal fluid (Fig. 8C), as well as increased neutrophil infiltration into the peritoneal cavity (Fig. 8D). To evaluate the longer-term effects of sustained high blood lactate levels during sepsis, CLP mice were treated with sodium lactate twice daily for four consecutive days (Fig. 8E). Notably, these mice exhibited a significant drop in body temperature (Fig. 8F) and increased mortality (Fig. 8G) compared to NaCl-treated controls. To determine whether these deleterious effects were mediated by NLRP3 inflammasome activation, sodium lactate–treated CLP mice were co-administered the selective NLRP3 inhibitor MCC950 (Fig. 8H) [47]. Pharmacological inhibition of NLRP3 markedly reduced IL-1β levels in both blood and peritoneal fluid (Fig. 8I), attenuated neutrophil recruitment to the peritoneal cavity (Fig. 8J), and significantly improved survival in sodium lactate–treated mice (Fig. 8K). These findings demonstrate that lactate-driven exacerbation of sepsis is largely dependent on NLRP3 inflammasome activity.

To exclude the possibility that these effects were attributable to nonspecific osmotic or sodium load, we performed comprehensive physiological analyses following sodium lactate or NaCl administration in CLP mice (Supplementary Table S1). Multivariate analysis revealed no overall treatment effect on systemic osmolarity, blood sodium concentration, or blood pH (Pillai’s Trace = 0.699, F = 3.094, *P* = 0.152). Univariate analyses showed that sodium lactate selectively increased blood pH compared with NaCl controls (7.245 ± 0.024 vs. 7.148 ± 0.024; *P* = 0.027), consistent with metabolic utilization of lactate as an alkalinizing substrate [48], whereas serum osmolarity (322.8 ± 8.2 vs. 316.5 ± 8.2 mOsm; *P* = 0.611) and blood sodium concentration (144.0 ± 3.3 vs. 144.8 ± 3.3 mM; *P* = 0.878) were unchanged between groups. These data indicate that sodium lactate administration does not impose additional osmotic or sodium burden. Collectively, these findings suggest that lactic acid production and accumulation exacerbate systemic inflammation and contribute to septic shock progression through NLRP3 inflammasome activation.

## Discussion

Glycolysis is essential for NLRP3 inflammasome activation [4, 10], yet the underlying mechanisms linking metabolic activity to inflammasome signaling have remained unclear. In this study, we demonstrate that endogenous lactic acid production following NLRP3 stimulation drives intracellular acidification, which promotes NLRP3 inflammasome activation, ASC oligomerization, and IL-1β secretion through a PKR-dependent mechanism. We further show that extracellular acidification impairs lactic acid efflux, thereby exacerbating intracellular acidification and amplifying inflammasome activation. Unexpectedly, lactic acid itself functions as a catalytic agent, directly cleaving pro-IL-1β at Asp116 to generate mature IL-1β. In vivo, elevated blood lactate levels enhance systemic IL-1β production and worsen sepsis outcomes in the CLP model. Together, these findings identify intracellular and extracellular lactic acidosis as key metabolic signals that regulate NLRP3 inflammasome activation and contribute to the pathogenesis of sepsis.

Activation of the NLRP3 inflammasome is accompanied by a marked increase in glycolytic flux in macrophages [11]. Inhibition of glycolysis—whether by glucose deprivation, downregulation of the glucose transporter GLUT1 [11], treatment with 2-deoxyglucose or PKM2 inhibitors [4, 11], or suppression of lactic acid fermentation via LDH inhibition with GSK2837808A [10]—consistently impairs inflammasome activation. These findings collectively demonstrate that aerobic glycolysis is essential for NLRP3 inflammasome activation. However, the mechanisms by which glycolysis-derived metabolites, particularly lactic acid, regulate this process have remained elusive. Here, we show that NLRP3 stimulation drives a glycolytic shift and increases lactic acid production in macrophages, leading to intracellular acidification. Blocking lactic acid accumulation or acidification—either through LDH inhibition or extracellular alkalinization—disrupts PKR–NLRP3 interaction, impairs ASC oligomerization, and ultimately suppresses inflammasome activation. While PKR has been previously implicated in NLRP3 inflammasome activation [14] and is well known for its role in sensing cytosolic double-stranded RNA to initiate antiviral responses [12], it also responds to metabolic stressors such as endoplasmic reticulum stress and oxidative stress [12, 13]. Our findings reveal a previously unrecognized function of PKR as a sensor of intracellular pH stress. Specifically, we propose that lactic acid—beyond serving as a terminal metabolite of glycolysis—acts as a signaling molecule that links metabolic activity to innate immune activation. By promoting intracellular acidification, lactic acid enables PKR engagement with NLRP3 and thereby facilitates inflammasome assembly. This establishes lactic acid as a critical metabolic-immune interface, offering new insights into how shifts in cellular metabolism regulate innate immune signaling. The role of PKR in NLRP3 inflammasome activation remains controversial, with some studies suggesting that PKR is dispensable in certain experimental contexts [49]. We propose that PKR involvement in NLRP3 signaling is stimulus- and context-dependent, becoming functionally relevant under conditions of pronounced intracellular lactic acid accumulation and acidification, which promote PKR phosphorylation and PKR–NLRP3 interaction. In settings lacking substantial metabolic reprogramming or intracellular pH stress, PKR may therefore appear non-essential. Further studies are required to define the precise conditions under which PKR contributes to NLRP3 inflammasome regulation.

Metabolic acidosis is involved in the pathogenesis of sepsis, type 2 diabetes, and chronic kidney disease by stimulating pro-inflammatory cytokine production, impairing glucose tolerance, and accelerating kidney disease progression, respectively [22]. In addition, the NLRP3 inflammasome contributes the development of these diseases [5, 50, 51]. However, the relationship between metabolic acidosis and NLRP3 inflammasome activation remains unclear. Our results demonstrate that extracellular acidic condition promotes intracellular lactic acidification, further enhancing NLRP3 inflammasome activation in macrophages through inhibiting lactic acid excretion. Although extracellular acidic condition alone does not activate the NLRP3 inflammasome, it enhances NLRP3 inflammasome activation in the presence of an NLRP3 activator. Consistent with our findings, Rajamäki et al. suggested that extracellular acidosis serves as a danger signal alerting innate immunity via the NLRP3 inflammasome, although the mechanism was not defined at that time [52]. Metabolic lactic acidosis is a condition characterized by rises in blood lactate (> 5 mM) and decreases in blood pH (< 7.25) [53]. It occurs in severe infections and is associated with the mortality in septic patients [44]. Our results show that extracellular acidic condition with a pH of 6.9, a level of acidity observed in septic patients [17], can enhance IL-1β secretion through NLRP3 inflammasome activation. Moreover, extracellular lactate treatment enhanced intracellular lactic acidification, NLRP3 inflammasome activation and IL-1β secretion only under acidic conditions, but not when applied alone, thereby exacerbating septic shock in the cecal ligation and puncture model. Consistent with our studies, NLRP3 knockout protects mice against endotoxic shock [5]. Our findings suggest a straightforward mechanism to explain how metabolic lactic acidosis exacerbates septic shock through NLRP3 inflammasome activation enhancement. Intracellular accumulation of lactic acid during acute glycolytic bursts promotes cytosolic acidification and NLRP3 inflammasome activation. In contrast, extracellular lactate has been shown to suppress pro-inflammatory responses through engagement of the cell-surface receptor GPR81 [54, 55]. On the other hand, metabolic acidosis with a pH of 6.9 was observed in the blood of diabetic ketoacidosis patients due to the rise of ketones [56] and in the tumor tissues of cancer patients due to the accumulation of lactic acid [57]. When the NLRP3 inflammasome is activated due to disease characteristics and participates in pathogenesis, our findings raised the possibility that metabolic acidosis may enhance intracellular lactic acidification, further promoting the activation of the NLRP3 inflammasome and thereby exacerbating these diseases. We therefore propose a biphasic model in which intracellular lactic acid supports early inflammatory responses during acute inflammation, whereas progressive extracellular lactate accumulation during prolonged inflammation acts as a negative-feedback mechanism to restrain excessive inflammasome activation in chronic inflammatory settings. Our work contributes to the understanding of the mechanisms by which acidic conditions enhance inflammatory diseases and may provide therapeutic targets for controlling inflammation and disease progression.

Glycolysis is a core energy-producing pathway in cells, converting glucose into two net ATPs and pyruvates. These pyruvates can then be utilized by the mitochondria to generate an additional 34 ATPs through mitochondrial oxidative phosphorylation. Concentrating glycolysis enzymes to form the glycolytic metabolon on the outer mitochondrial membrane [58] reduces the diffusion distance of glycolytic intermediates, thereby enhancing rates of glucose utilization for mitochondrial oxidative phosphorylation and fermentative glycolysis [59]. Lactic acid is the end product of fermentative glycolysis. The considerable production of lactic acid may cause local intracellular acidification [60], particularly near the outer mitochondrial membrane, which is the location of the glycolytic metabolon. Notably, the NLRP3 inflammasome protein complex co-localizes with mitochondria [61]. These findings prompt us to hypothesize that in response to NLRP3 stimulation, the increase in lactic acid production causes lactic acidification near the mitochondria. This lactic acidification regulates NLRP3 inflammasome formation. In addition, during NLRP3 inflammasome activation, a subset of macrophages undergoes marked cytosolic acidification, reaching pH values as low as 4.1, comparable to conditions under which lactic acid–mediated pro–IL-1β cleavage is observed in cell-free assays. These findings raise the possibility that severe, localized intracellular acidification may permit direct lactic acid–mediated cleavage of pro–IL-1β within cells. In IL-1β regulation pathway, IL-1β secretion is tightly controlled by NLRP3 inflammasome-dependent caspase-1 activation [3]. The inactive precursor pro-IL-1β is not secretable and accumulates within cells until it is cleaved by caspase-1 at Asp116 to form two smaller proteins [62]. The carboxyl-terminal part is a mature IL-1β protein, which is secretable and has a higher binding affinity to its surface receptor IL-1R1 compared to its precursor pro-IL-1β protein [2]. The engagement of mature IL-1β and IL-1R1 is required to activate the inflammatory signals [62]. Our findings suggest that lactic acid is a pro-inflammatory metabolite that promotes IL-1β secretion through both inflammasome-dependent and -independent mechanisms.

Here, we identify lactic acid as a previously unrecognized mediator that directly cleaves pro–IL-1β at Asp116, mimicking the enzymatic activity of caspase-1. Under equivalent acidic conditions, organic carboxylic acids such as lactic acid and acetic acid exhibit markedly greater cleavage efficiency than HCl, suggesting that the catalytic activity is conferred not solely by proton concentration, but by intrinsic chemical properties of the carboxyl group. Supporting this, we observed a positive correlation between mature IL-1β production and the proportion of lactate converted to lactic acid across a range of pH values. Aspartic acid, an acidic amino acid with a side-chain pKa of 3.9, undergoes preferential hydrolysis at aspartyl peptide bonds under mildly acidic conditions that preserve the undissociated β-carboxyl group. We therefore propose a non-enzymatic cleavage mechanism involving intramolecular anhydride or cyclic imide intermediates formed via condensation between the β-carboxyl and adjacent amide groups [63]. This model is supported by the loss of detectable IL-1β cleavage at pH values above 4 (Fig. 6C), and by prior reports demonstrating that formic acid, a structurally simpler organic acid, induces site-specific cleavage at aspartyl residues [63]. Formic acid–based chemical cleavage has been widely used for peptide mapping by mass spectrometry. Our findings extend this principle, revealing that lactic acid—a physiologically abundant metabolite—possesses similar site-selective catalytic potential. This previously unappreciated function of lactic acid defines a new class of non-enzymatic, site-specific chemical modifications with broad implications for synthetic biology. In particular, the ability of carboxylic acids to mediate aspartyl bond hydrolysis may enable the development of biomimetic catalysts, nanozymes, and acid-responsive systems for conditional control of protein function in nanobiotechnology and protein engineering.

In summary, our research elucidates the mechanisms by which glycolysis triggers NLRP3 inflammasome activation. Upon NLRP3 stimulation, glycolysis activation produces lactic acid, further inducing intracellular acidification, which triggers PKR-NLRP3 protein interaction and in turn promotes NLRP3 inflammasome formation and IL-1β secretion. Extracellular acidification further enhances this process by hindering lactic acid excretion. Notably, lactic acid itself can directly cleave the precursor pro-IL-1β to generate mature IL-1β, highlighting a novel enzymatic role of lactic acid in chemical cleavage and inflammatory response. These findings explain how metabolic acidosis exacerbates inflammatory diseases and could inform therapeutic strategies for metabolic acidosis diseases like sepsis. Furthermore, our results uncover a previously unrecognized role for PKR as a stress-responsive mediator linking intracellular metabolic acidification to inflammasome activation, adding a new layer to the immunometabolic regulation of inflammation.

## Supplementary information

Reproducibility checklist

Supplementary Figures

Supplementary Table S1

Western blot original films

## Data Availability

The datasets used and analyzed in this study are available from the corresponding authors on reasonable requests.
