# Supplementary material for: Lactic acid drives NLRP3 inflammasome activation and caspase-1–like cytokine cleavage via intracellular acidification
Source: Cell Death Dis. 2026 Apr 3;17(1):450. doi: 10.1038/s41419-026-08708-y (PMC13172327; doi:10.1038/s41419-026-08708-y)
Supplement: Supplementary file 2 — Supplementary Figures [file 41419_2026_8708_MOESM2_ESM.docx]

**Supplementary Information**

**Lactic acid drives NLRP3 inflammasome activation and caspase-1–like cytokine cleavage via intracellular acidification**

Hsin-An Lin, Hsin-Chung Lin, Ming-Hang Tsai, Yu-Jen Chen, Bo-Ying Bao, Kuen-Jou Tsai, Chieh-Tien Shih, Jau-Song Yu, Kun-Yi Chien, Kuo-Yang Huang, David M. Ojcius, and Lih-Chyang Chen

**Supplementary Contents**

**Supplementary Figures and Figure Legends**

**Supplementary Fig. S1.** Pharmacological modulation of GPR81 does not alter extracellular lactic acid–enhanced NLRP3 inflammasome activation.

**Supplementary Fig. S2.** Inhibition of lactate dehydrogenase suppresses AIM2 inflammasome activation.

**Supplementary Fig. S3.** Extracellular lactic acid–induced intracellular acidification does not alter potassium efflux during NLRP3 inflammasome activation.

**Supplementary Fig. S1**


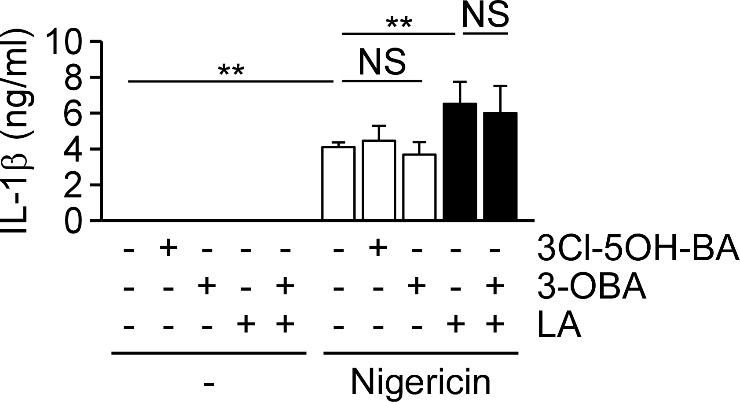


**Supplementary Fig. S1. Pharmacological modulation of GPR81 does not alter extracellular lactic acid–enhanced NLRP3 inflammasome activation.** THP-1-derived macrophages were stimulated with nigericin in the presence or absence of extracellular lactic acid (10 mM), the GPR81 antagonist 3-OBA (15 mM), or the GPR81 agonist 3Cl-5OH-BA (1 mM). IL-1β levels in culture supernatants were quantified by ELISA (n = 3). Data are presented as mean ± SD. Statistical significance was determined by one-way ANOVA with Tukey’s HSD post hoc test; NS, not significant; **P < 0.01.

**Supplementary Fig. S2**


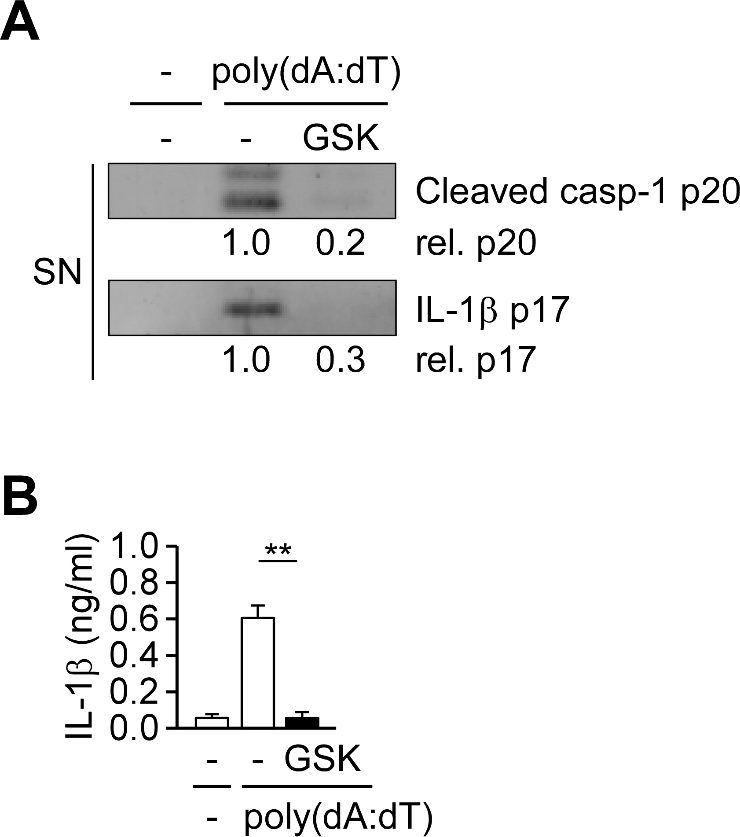


**Supplementary Fig. S2. Inhibition of lactate dehydrogenase suppresses AIM2 inflammasome activation.** (A) Immunoblot analysis of caspase-1 p20 and IL-1β p17 in culture supernatants of THP-1-derived macrophages stimulated with poly(dA:dT) in the presence or absence of the lactate dehydrogenase inhibitor GSK2837808A (10 μM). The western blot is a representative of three independent experiments. (B) ELISA quantification of IL-1β secretion from THP-1-derived macrophages treated as in (A) (n = 4). Data are presented as mean ± SD. Statistical significance was assessed by one-way ANOVA with Tukey’s HSD post hoc test; **P < 0.01.

**Supplementary Fig. S3**


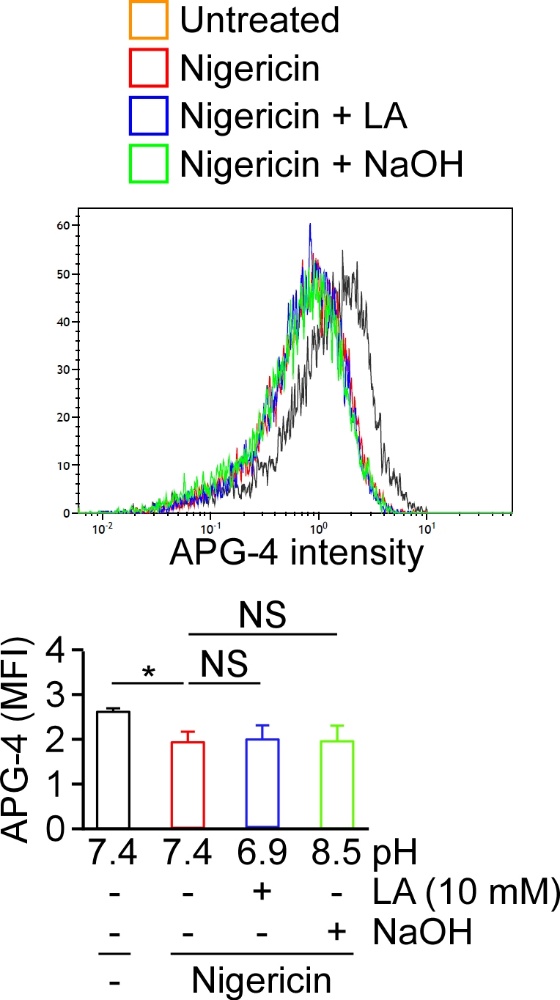


**Supplementary Fig. S3. Extracellular lactic acid–induced intracellular acidification does not alter potassium efflux during NLRP3 inflammasome activation.** THP-1-derived macrophages were stimulated with nigericin in the presence or absence of extracellular lactic acid (LA, 10 mM; pH 6.9) or extracellular alkalinization (NaOH; pH 8.5) for 8 min. Intracellular potassium levels were quantified by APG-4 fluorescence using flow cytometry (n = 3). Data are presented as mean ± SD and were analyzed by one-way ANOVA with Tukey’s HSD post hoc test; *P < 0.05; NS, not significant.
