# Supplementary material for: Lactic acid drives NLRP3 inflammasome activation and caspase-1–like cytokine cleavage via intracellular acidification
Source: Cell Death Dis. 2026 Apr 3;17(1):450. doi: 10.1038/s41419-026-08708-y (PMC13172327; doi:10.1038/s41419-026-08708-y)
Supplement: Supplementary file 3 — Supplementary Table S1 [file 41419_2026_8708_MOESM3_ESM.docx]

| **Supplementary Table S1.** Sodium lactate selectively increases blood pH without altering osmolarity or sodium concentration in CLP mice | | | | | |
| --- | --- | --- | --- | --- | --- |
| Parameter | Treatment | Mean ± SEM | 95% CI | F | P |
| Osmolarity (mOsm) | NaCl | 316.500 ± 8.230 | 296.361 – 336.639 | 0.288 | 0.611 |
|  | NaL | 322.750 ± 8.230 | 302.611 – 342.889 |  |  |
| pH | NaCl | 7.148 ± 0.024 | 7.090 – 7.205 | 8.529 | 0.027* |
|  | NaL | 7.245 ± 0.024 | 7.187 – 7.303 |  |  |
| Na^+^ (mM) | NaCl | 144.750 ± 3.321 | 136.623 – 152.877 | 0.025 | 0.878 |
|  | NaL | 144.000 ± 3.321 | 135.873 – 152.127 |  |  |

Cecal ligation and puncture (CLP) mice were administered sodium chloride (NaCl) or sodium lactate (NaL) 0.5 h after CLP surgery (n = 4 per group). Blood osmolarity, pH, and sodium concentration were measured 6 h after CLP. Data were analyzed using a multivariate general linear model with treatment as the fixed factor. Multivariate significance was assessed using Pillai’s Trace. Data are presented as estimated marginal means ± SEM with 95% confidence intervals (CI). F statistics and P values are reported for univariate between-group effects. * With statistic significance.
