# Supplementary material for: Lactic acid drives NLRP3 inflammasome activation and caspase-1–like cytokine cleavage via intracellular acidification
Source: Cell Death Dis. 2026 Apr 3;17(1):450. doi: 10.1038/s41419-026-08708-y (PMC13172327; doi:10.1038/s41419-026-08708-y)

Figure 1

Fig. 1B

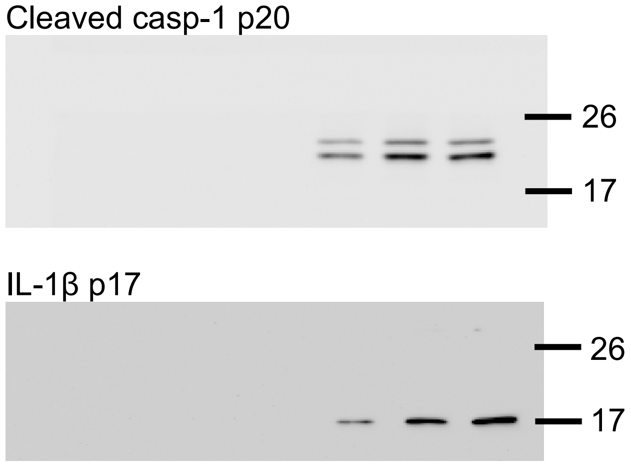

Fig. 1D

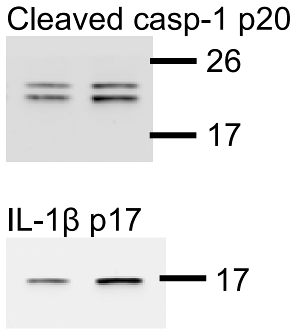

Fig. 1C

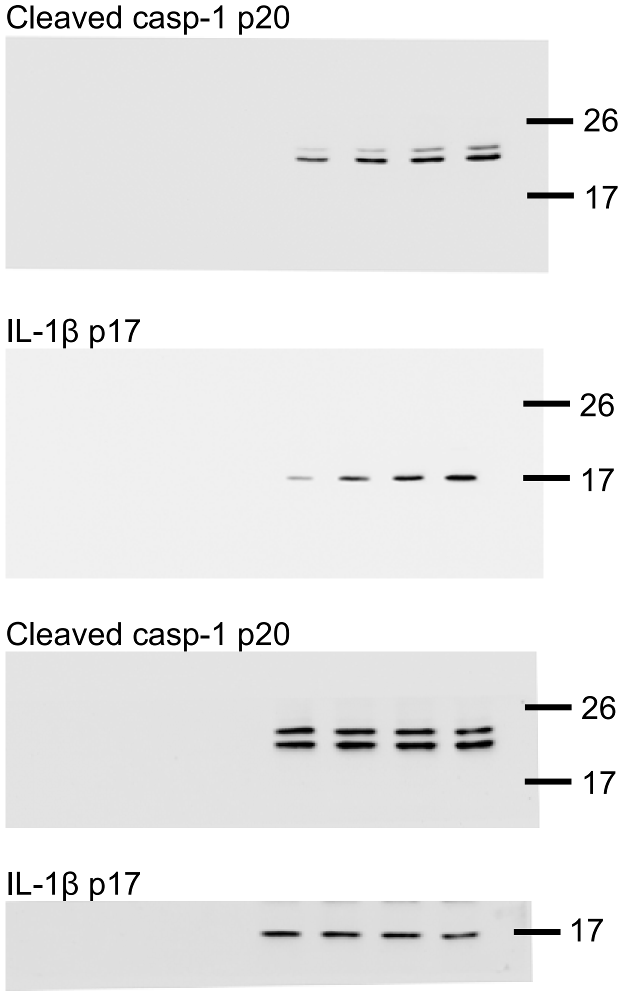

Figure 2

Fig. 2C

Cleaved casp-1 p20

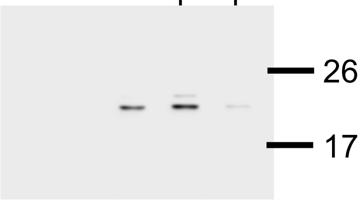

IL-1β p17

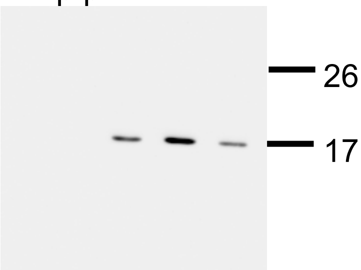

Figure 3

Fig. 3I

Cleaved casp-1 p20

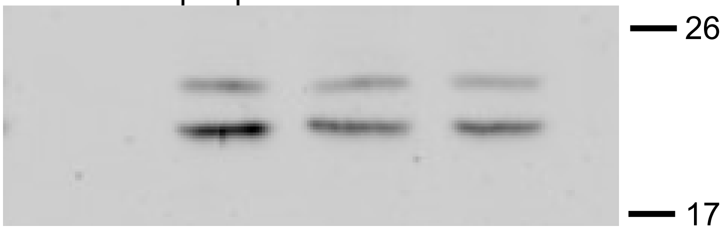

IL-1β p17

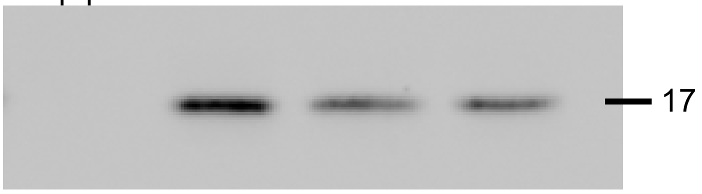

Figure 4

Fig. 4D

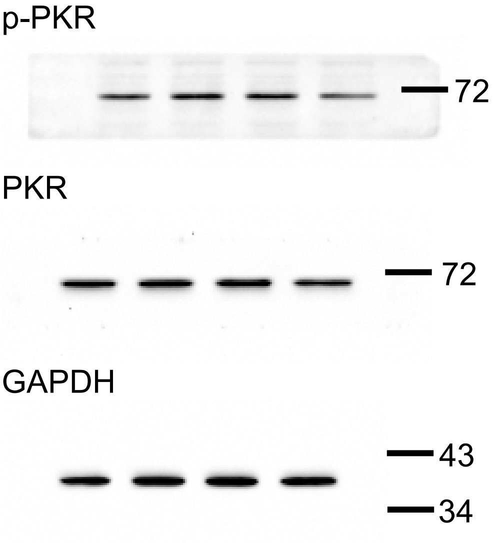

Fig. 4E

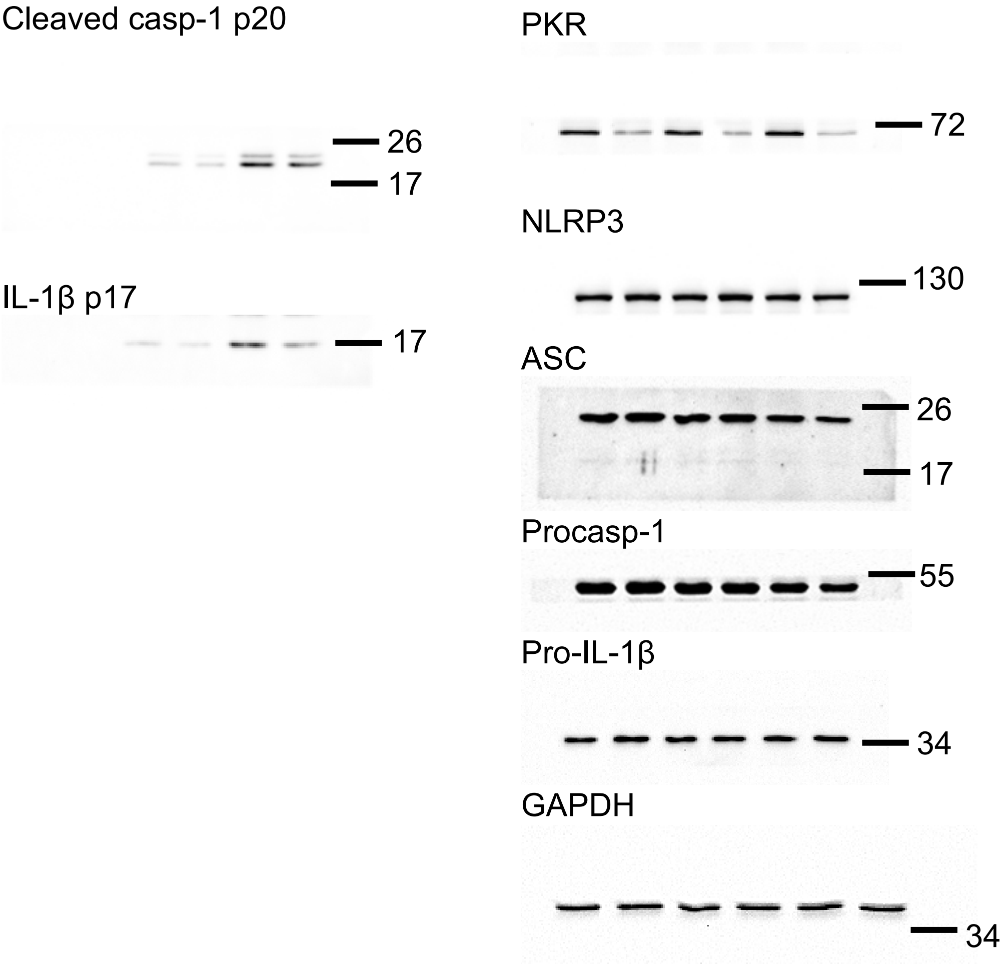

Fig. 4G

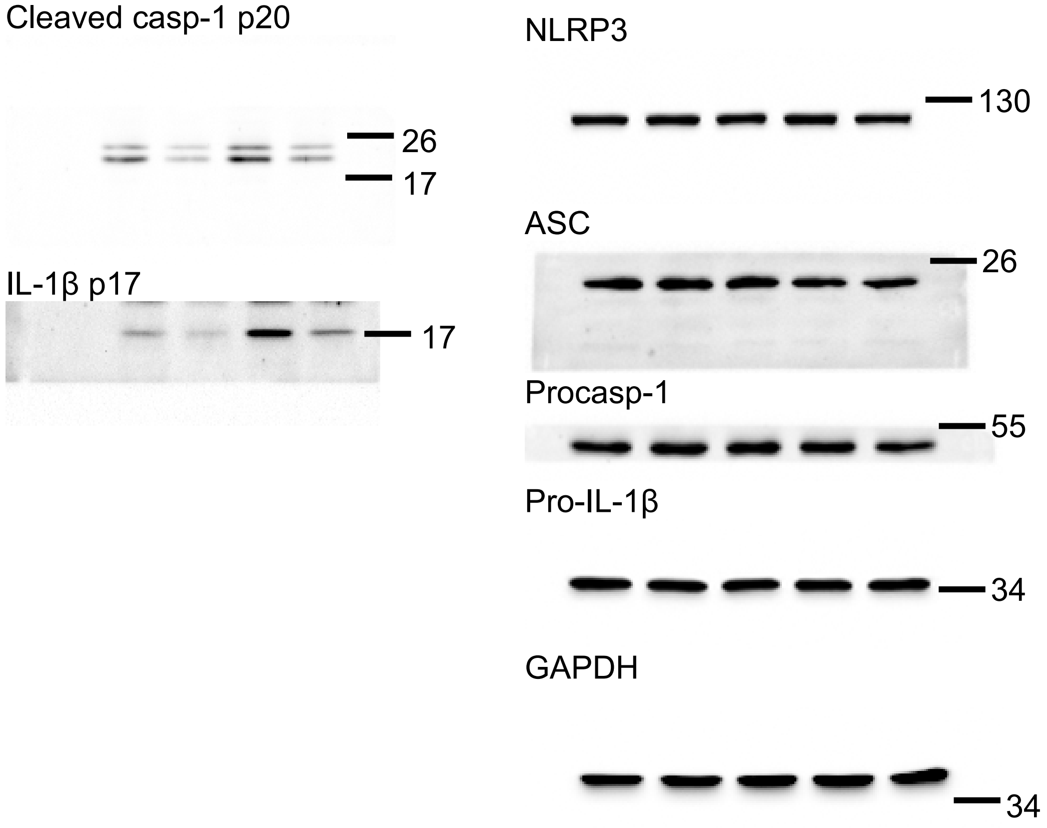
Cleaved casp-1 p20
26
17
IL-1β p17
17
NLRP3
130
ASC
26
Procasp-1
55
Pro-IL-1β
34
GAPDH
34

Fig. 4I

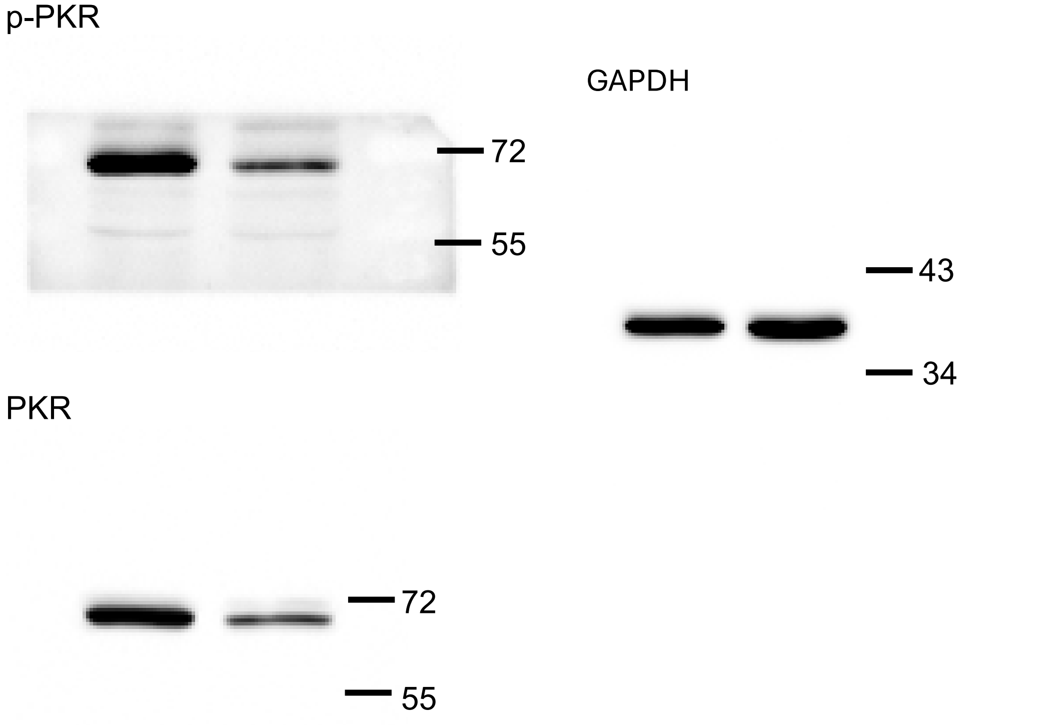
p-PKR
72
55
GAPDH
43
34
PKR
72
55

Fig. 4J

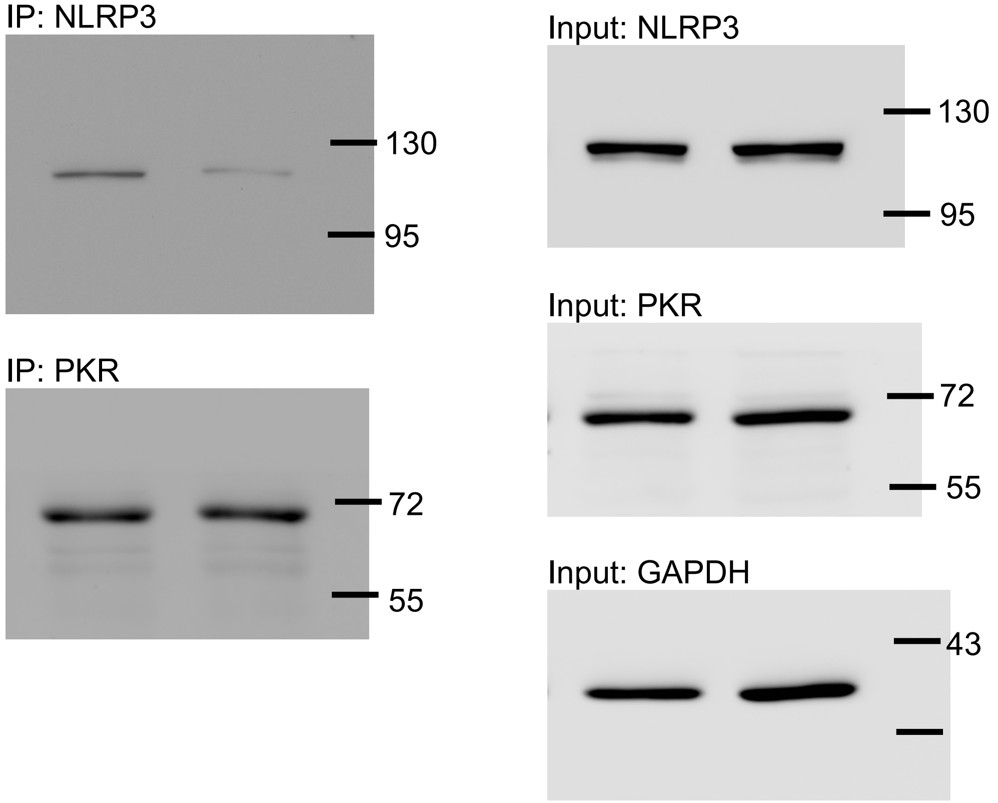
IP: NLRP3
130
95
IP: PKR
72
55
Input: NLRP3
130
95
Input: PKR
72
55
Input: GAPDH
43

Fig. 4K

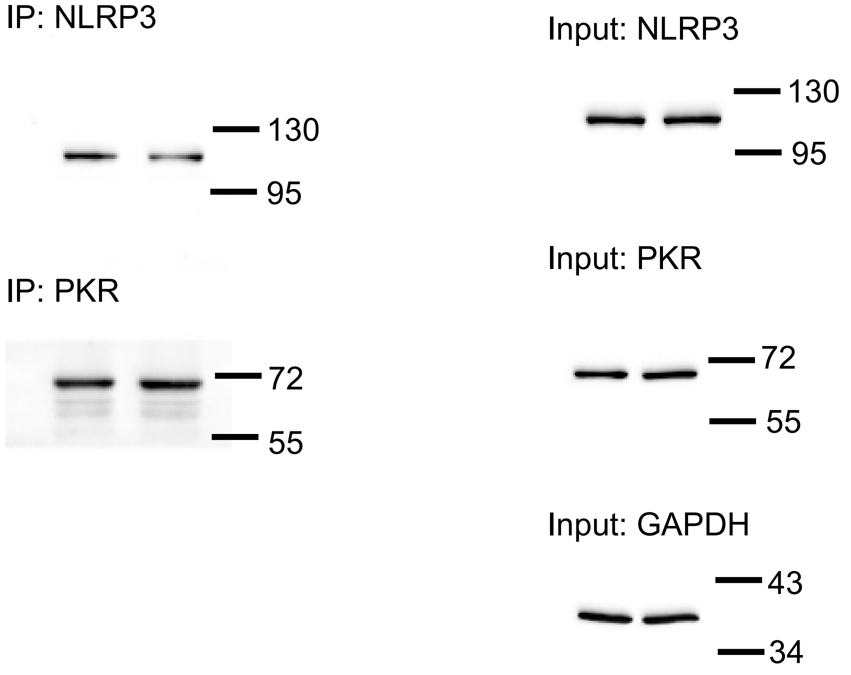
IP: NLRP3
130
95
IP: PKR
72
55
Input: NLRP3
130
95
Input: PKR
72
55
Input: GAPDH
43
34

Figure 5

Fig. 5B

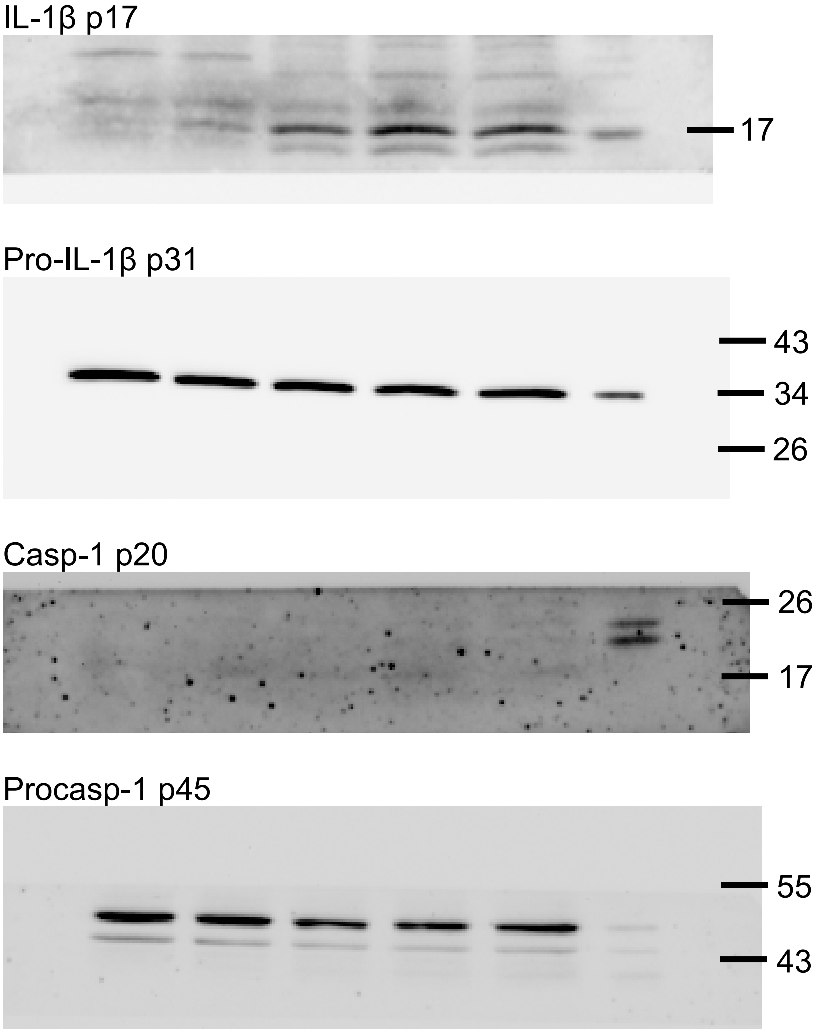

Fig. 5C

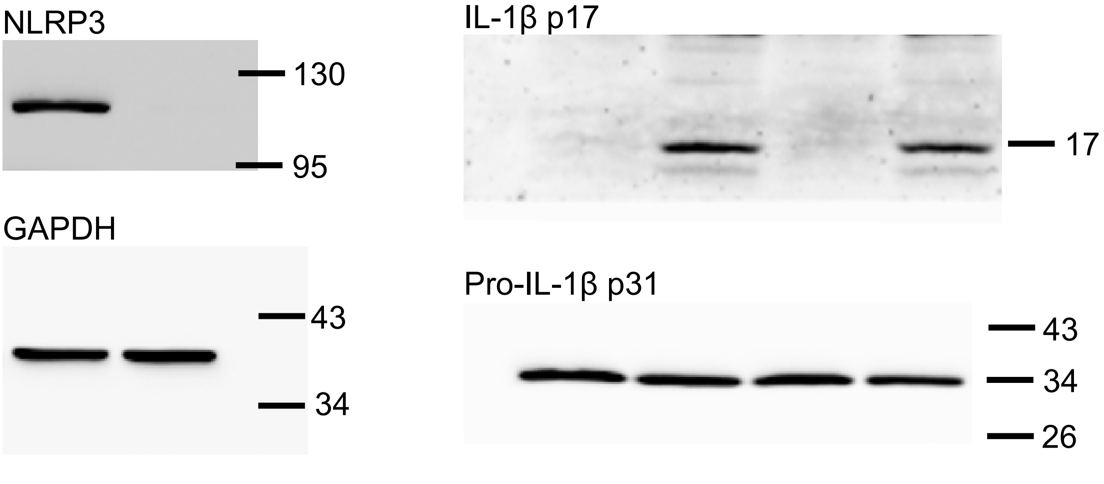

Fig. 5D

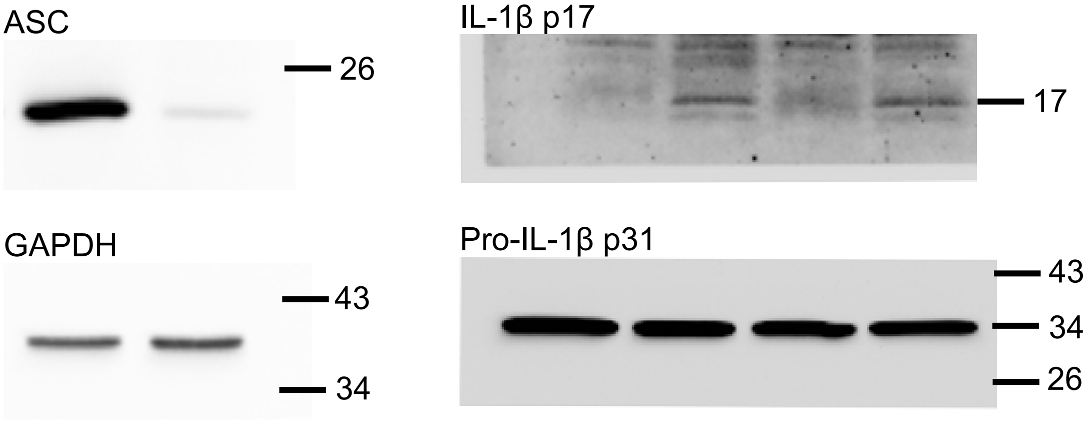

Fig. 5E

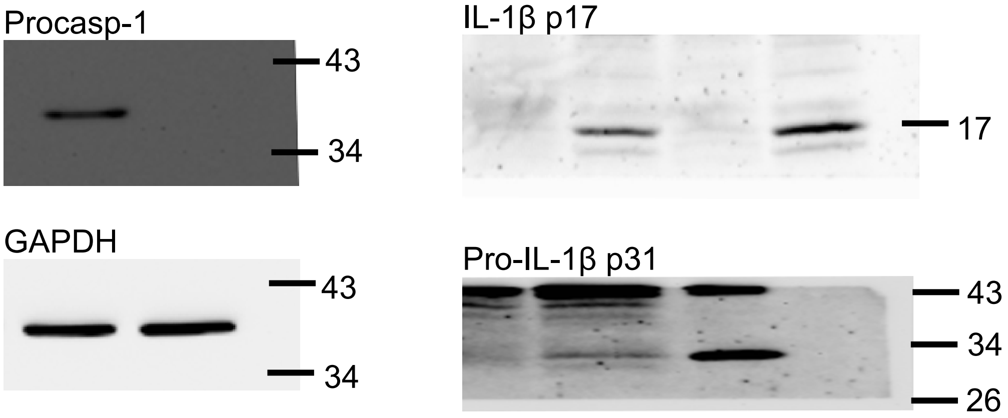

Fig. 5F

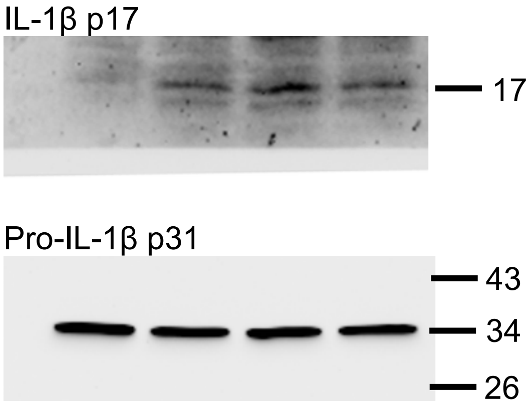

Fig. 5G

IL-1β p17

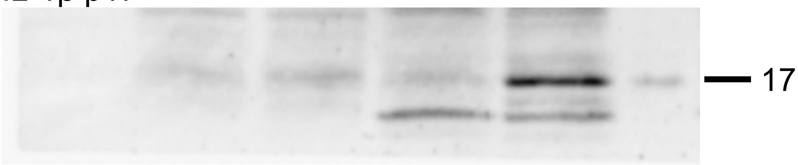

Pro-IL-1β p31

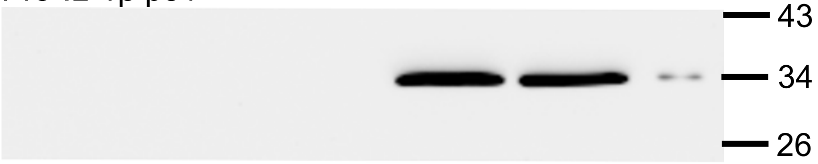

Fig. 5H

IL-1β p17

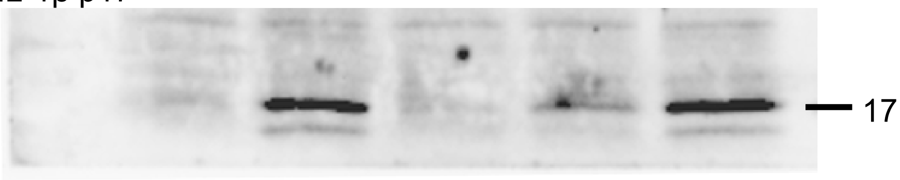

Pro-IL-1β p31

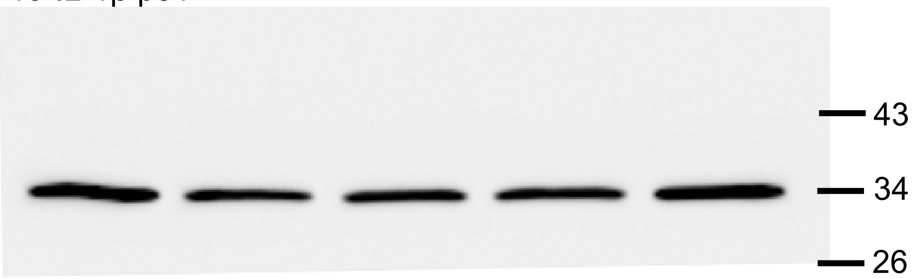

Figure 6

Fig. 6A

Pro-IL-1β-FLAG

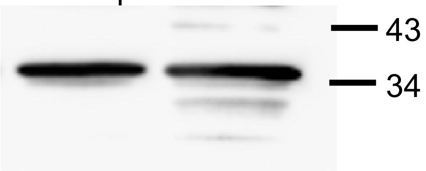

IL-1β-FLAG

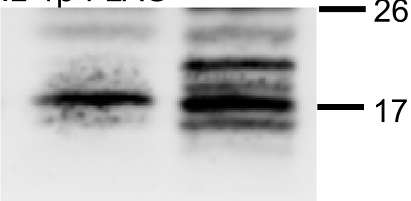

Fig. 6B

Pro-IL-18 / IL-18

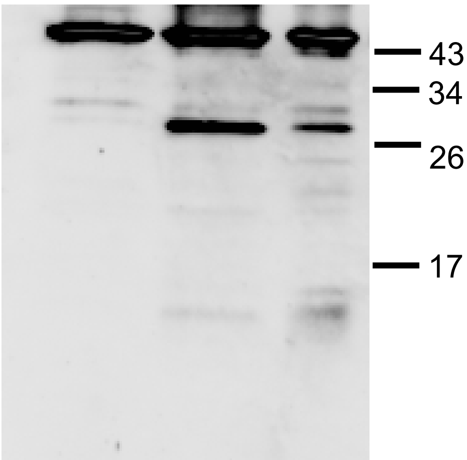

Fig. 6C

Pro-IL-1β / IL-1β

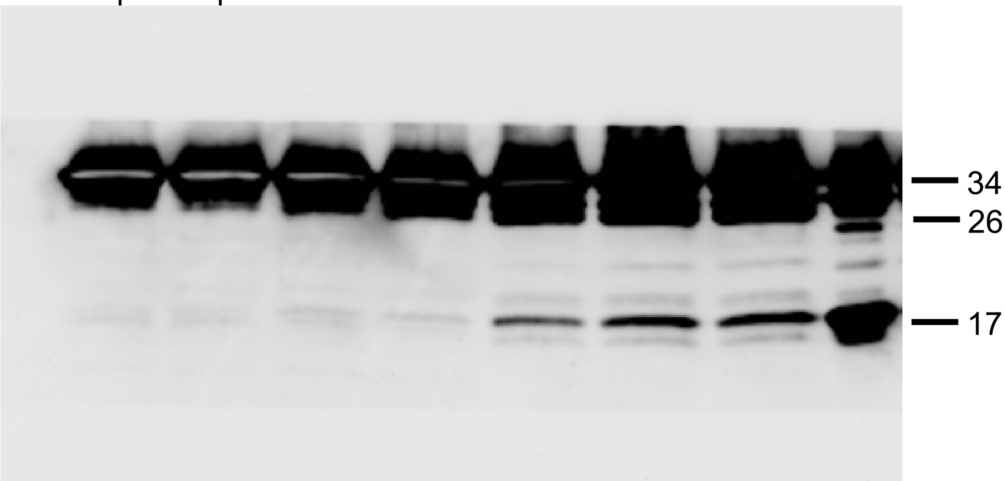

Fig. 6D

Pro-IL-1β / IL-1β

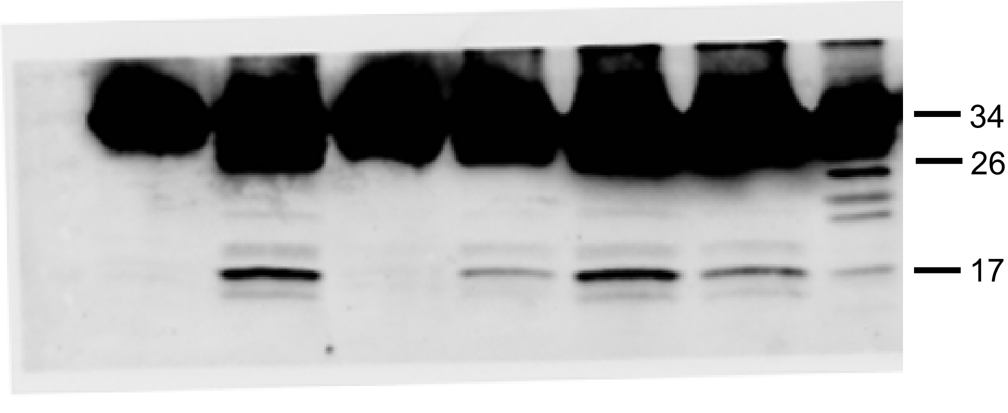

Figure 7

Fig. 7B

IL-1β

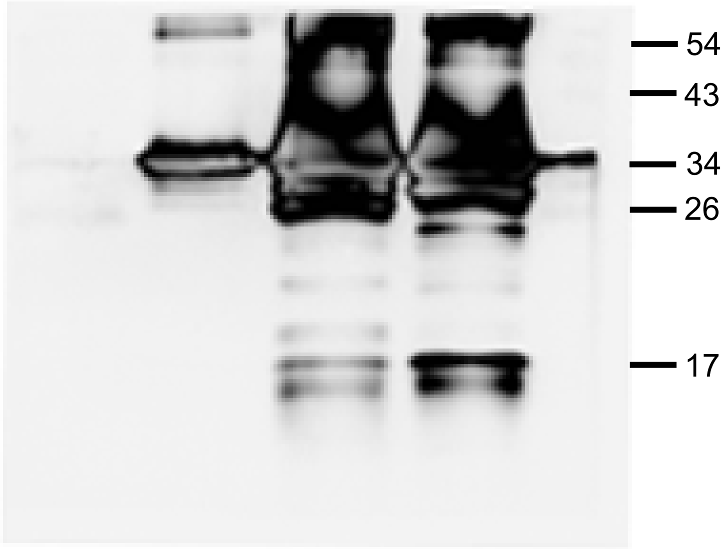

Fig. 7D

Pro-IL-1β-FLAG / IL-1β-FLAG

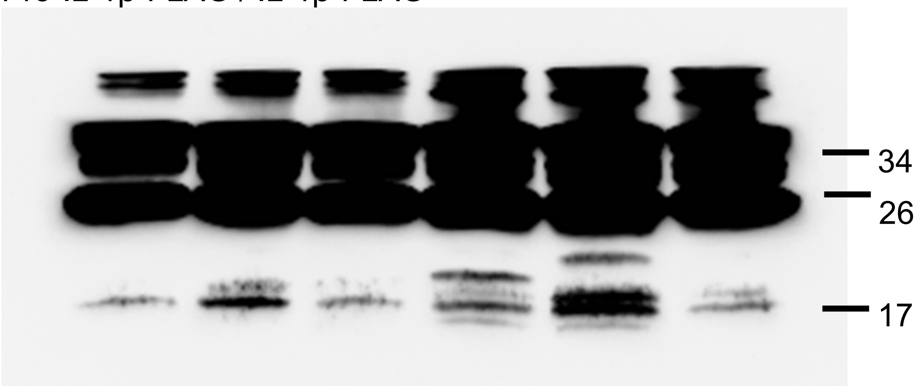

Supplementary Figure S2

Fig. S2A

Cleaved casp-1 p20

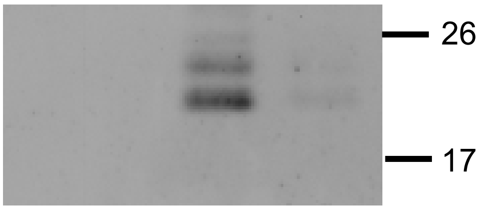

IL-1β p17

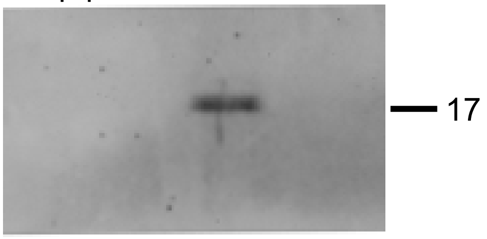

Supplement: Supplementary file 4 — Western blot original films [file 41419_2026_8708_MOESM4_ESM.pdf]
